# Exploring mechanisms underlying heterospecific alarm call responses in meerkats and yellow mongooses

**DOI:** 10.1093/beheco/arag031

**Published:** 2026-04-03

**Authors:** Nikola Falk, Stuart K Watson, Vanessa Rüegg, Marta B Manser

**Affiliations:** Department of Evolutionary Biology and Environmental Studies, University of Zurich, Winterthurerstrasse 190, Zurich 8057, Switzerland; Kalahari Research Centre, Kuruman River Reserve, Van Zylsrus 8467, South Africa; Institute for Interdisciplinary Study of Language Evolution, ISLE, University of Zurich, Affolternstrasse 56, Zurich 8050, Switzerland; Department of Evolutionary Biology and Environmental Studies, University of Zurich, Winterthurerstrasse 190, Zurich 8057, Switzerland; Institute for Interdisciplinary Study of Language Evolution, ISLE, University of Zurich, Affolternstrasse 56, Zurich 8050, Switzerland; Department of Comparative Language Science, University of Zurich, Affolternstrasse 56, Zurich 8050, Switzerland; Department of Evolutionary Anthropology, University of Zurich, Winterthurerstrasse 190, Zurich 8057, Switzerland; Department of Evolutionary Biology and Environmental Studies, University of Zurich, Winterthurerstrasse 190, Zurich 8057, Switzerland; Kalahari Research Centre, Kuruman River Reserve, Van Zylsrus 8467, South Africa; Institute for Interdisciplinary Study of Language Evolution, ISLE, University of Zurich, Affolternstrasse 56, Zurich 8050, Switzerland; Department of Evolutionary Biology and Environmental Studies, University of Zurich, Winterthurerstrasse 190, Zurich 8057, Switzerland; Kalahari Research Centre, Kuruman River Reserve, Van Zylsrus 8467, South Africa; Institute for Interdisciplinary Study of Language Evolution, ISLE, University of Zurich, Affolternstrasse 56, Zurich 8050, Switzerland

**Keywords:** heterospecific alarm calls, eavesdropping, vocal signal evolution, alarm calling, vocal communication

## Abstract

“Eavesdropping” on heterospecific alarm calls is common, but we are only beginning to understand the cognitive mechanisms involved. A key question is whether eavesdropping results from innate perceptual sensitivities to acoustic features that are common to the calls of diverse species, or if it is a learned behavior. Here, we used playback experiments to investigate whether wild and captive meerkats (*Suricata suricatta*), and wild yellow mongooses (*Cynictis penicillata*) respond in a functionally appropriate way, ie successfully “eavesdrop”, to heterospecific alarm calls. We tested calls from both sympatric and allopatric species to reveal whether responses were a result of learned associations between call types and their referents, or of innate perceptual sensitivities to their acoustic features. By playing back calls which signal either “aerial” or “general” predation threats, we also explored whether subjects could differentiate between the underlying function of heterospecific alarm calls. Our results show that wild meerkats and wild yellow mongooses, but not captive meerkats, were more likely to respond to the alarm calls of sympatric species. Additionally, wild, but not captive, meerkats were also more likely to flee as a response to aerial as opposed to general alarm calls. This indicates that learning likely affects the response of mongooses to heterospecific alarm calls. However, other factors are also likely to have an impact on how individuals in different populations of the same species respond to heterospecific alarm calls, such as innate perceptual sensitivities, external threats, the need to respond, and the soundscape a species is exposed to.

## Teaser

To explore how species “eavesdrop” on the alarm calls of others, we played alarm calls from familiar and unfamiliar species to wild meerkats, yellow mongooses, and captive meerkats. Our results indicated that all three groups recognized the sounds as alarm calls, but processed them differently. Only wild meerkats behaved in a way that was consistent with understanding whether the calls referred to an aerial or general threat, indicating eavesdropping is somewhat dependent on learning processes.

## Introduction

Many species produce alarm calls when detecting predators or threats in their environment to warn conspecifics about impending danger, making the production and perception of alarm calls important for a species' survival. Due to the important communicative functions of alarm calls, they have served as a classical model to study how different factors, such as the immediate behavioral context and motivational state of the caller, impact the acoustic structure of the calls (Marler 1955; Marler 1956; Morton 1977; Magrath et al. 2020). In addition to these caller-specific factors, how receivers process the acoustic features of a call also impact the evolution of vocalizations (ten Cate and Rowe 2007). For instance, the frightening or attention-grabbing acoustic features associated with alarm calls across species, such as nonlinear phenomena and deterministic chaos, may be adaptive because they prevent receivers from habituating to the calls, or make receivers more attentive (Fitch et al. 2002). Shared mechanisms underlying the perception and processing of alarm calls have been argued to lead to convergent evolution in the acoustic features of alarm calls across distantly related taxa (Marler 1955; Marler 1956).

In addition to the processing of conspecific alarm calls, some species are able to infer the presence of a predator from the alarm calls of heterospecifics by eavesdropping. Eavesdropping on heterospecific alarm calls is widespread in the animal kingdom, but the mechanisms underlying this phenomenon are not fully understood (Magrath et al. 2015a, 2015b; Magrath et al. 2020; Turner et al. 2023). One possibility is that the heterospecific eavesdroppers possess perceptual sensitivities to specific acoustic features (potentially the same as those of the signaler species) which allow them to respond to heterospecific alarm calls in a functionally appropriate way, without prior experience of the calls (Magrath et al. 2015a, 2015b). For instance, naïve nestlings of two sympatric Paridae species (*Parsus minor* and *Sittiparus varius*) fledge from their nest in response to the other species' “snake” alarm call. This behavior seems to result from acoustic commonalities between the calls of the two species (Ha et al. 2023). The processing of heterospecific calls is not limited to alarm calls indicating a predator attack. It has also been found that apostle birds (*Struthidea cinerea)* show a significantly higher mobbing response to the mobbing calls of heterospecific allopatric species than to control stimuli (Johnson et al. 2003), indicating an understanding of the alarm call function of these calls despite a lack of prior exposure. Furthermore, the acoustic features of infant distress calls that elicit a response from caregivers are shared across diverse mammalian species. For instance, white-tailed deer (*Odocoileus virginianus*) and mule deer (*Odocoileus hemionus*) respond to heterospecific infant distress calls of several mammalian species without prior exposure (Teichroeb et al. 2013; Lingle and Riede 2014). Moreover, humans can recognize the arousal state expressed by a variety of call types from distantly related taxa when played back their vocalizations, indicating acoustic universals that encode emotional states across terrestrial vertebrates (Filippi et al. 2017). Therefore, the ability to extract information from the calls of heterospecifics based on common acoustic cues is evidently widespread for various call types, presenting one plausible explanation for heterospecific eavesdropping of alarm calls.

However, in comparison to infant distress calls, the acoustic structures of alarm calls given as a response to predator attacks are highly diverse across species (Wheatcroft and Price 2013; Magrath et al. 2015a, 2015b). This raises the question as to whether heterospecific eavesdropping can solely be explained by common acoustic features and shared perceptual sensitivities. Learning has been shown to play a role in the recognition of heterospecific alarm calls in some species. For example, bonnet macaques (*Macaca radiata*) only respond to the playbacks of alarm calls of Nilgiri langurs (*Trachypithecus johnii*) and Hanuman langurs (*Semnopithecus entellus*) if they are frequently exposed to these species in their natural habitat (Ramakrishnan and Coss 2000). There is also experimental evidence that superb fairy wrens (*Malurus cyaneus*) can quickly learn to associate predator cues with novel heterospecific alarm calls (Magrath et al. 2015b). Studying whether different species respond to heterospecific alarm calls in functionally appropriate ways due to innate perceptual sensitivities, or if the association between heterospecific calls and their functions are learned, can grant us insight into the mechanisms underlying eavesdropping.

Across species, alarm calls fulfill a broad range of specific functions (Klump and Shalter 1984; Shelley and Blumstein 2005; Furrer and Manser Marta 2009a, 2009b; Zuberbühler 2009; Magrath et al. 2015a). For example, many species produce “general” alarm calls (Dezecache and Berthet 2018) in response to diverse types of threat, the acoustics of which may vary on an urgency gradient (eg banded mongooses, *Mungos mungo*, Jansen 2013; yellow mongooses, *Cynictis penicillata*, Le Roux et al. 2009; Cape ground squirrels, *Xerus inauris,* Furrer and Manser Marta 2009a, 2009b). Meanwhile, alarm calls can also be highly specific to the type of predator encountered (eg vervet monkeys, *Chlorocebus aethiops*, Seyfarth et al. 1980), or both predator type and perceived urgency level (eg meerkats, *Suricata suricatta*, Manser 2001). In some cases, heterospecifics can extract specific information on the type of predation threat eliciting the alarm call. For instance, Diana monkeys (*Cercopithecus diana*) produce different alarm calls when encountering crowned eagles (*Stephenoaetus coronatus*) and leopards (*Panthera pardus*) (Rainey et al. 2004). Yellow-casqued hornbills (*Ceratogymna elata*), which are predated by crowned eagles, but not leopards, can correctly distinguish between these calls (Rainey et al. 2004). This example is striking due to the phylogenetic distance between the signalers and their eavesdroppers. Heterospecific eavesdropping is clearly a highly adaptive behavior, allowing species to improve their defense against predation at minimal cost. However, the processes underlying this behavior in each case are largely unknown.

Two plausible and nonmutually exclusive mechanisms underlying heterospecific eavesdropping are: (i) an innate perceptual sensitivity to the acoustics of the calls of the signaling species, and (ii) learned associations through exposure to the call type and its eliciting context. In the case of innate perceptual sensitivity to the signaling species' alarm calls, this may be the simple result of an overlap in the acoustic features of the two species' alarm calls. For instance, “aerial flee” alarm calls, which are elicited by aerial predators and provoke a flee response in receivers, are, often associated with a narrow-band, high-pitched acoustic structure, which makes the source of the call difficult for predators to locate (Marler 1955; Marler 1956; Jones and Hill 2001; Magrath et al. 2015a, 2015b). In these cases of acoustic similarity between call types of similar function, the eavesdroppers may experience similar arousal states in response to the signals of both conspecific and heterospecific alarm calls. An innate response could also take place in the absence of acoustic similarity, because eavesdropping may be facilitated by the eavesdropping species having undergone selection to attend to the specific acoustic features of the signaling species. Finally, while the ability to make learned associations between heterospecific alarm calls and their referents is arguably a more cognitively taxing means of eavesdropping, it would provide receivers with the flexibility to quickly adapt to variability in the heterospecific species present. Exploring these hypotheses will grant us a better understanding of the evolution of this highly adaptive antipredator behavior.

The Herpestidae family is particularly interesting to explore these questions, because of the large variation of alarm call repertoires between closely related species (Manser 2001; Manser et al. 2014; Collier et al. 2017). Meerkats and dwarf mongooses, both cooperatively breeding species, have extensive and highly differentiated alarm repertoires (Manser 2001; Manser et al. 2014; Collier et al. 2017). Yellow mongooses, which primarily forage solitarily, but sometimes reunite with other conspecifics, eg when rearing offspring, (Manser et al. 2014), only produce a general alarm which does not differ between predator types, but may vary in its acoustic structure depending on the urgency of the threat (Le Roux et al. 2009). Additionally, despite being group living and cooperative, banded mongooses only produce a general alarm call and do not differentiate between predator types (Jansen 2013). Some mongoose species are known to eavesdrop on the alarm calls of sympatric species. For example, meerkats are more likely to abandon their food source as a response to alarm calls from fork-tailed drongos *(Dicrurus adsimilis*) and glossy starlings (*Lamprotornis nitens*) than to nonalarm calls of these species (Flower 2011). Banded mongooses respond to the alarm calls of sympatric plover species (*Vanellus spp.*), but fail to distinguish between the urgency levels of these alarm calls (Müller and Manser 2008). Dwarf mongooses respond to the alarm calls of tree squirrels (*Paraxerus cepapi*), who are predated by the same species as themselves, but ignore the alarm calls of chacma baboons (*Papio ursinus*), which are predated by different species (Morris-Drake et al. 2017). However, it is unknown whether these examples of interspecies eavesdropping are the result of innate perceptual sensitivities to certain acoustic properties of alarm calls, or whether mongooses instead learn the alarm calls of the species in their immediate home range.

**Figure 1:**
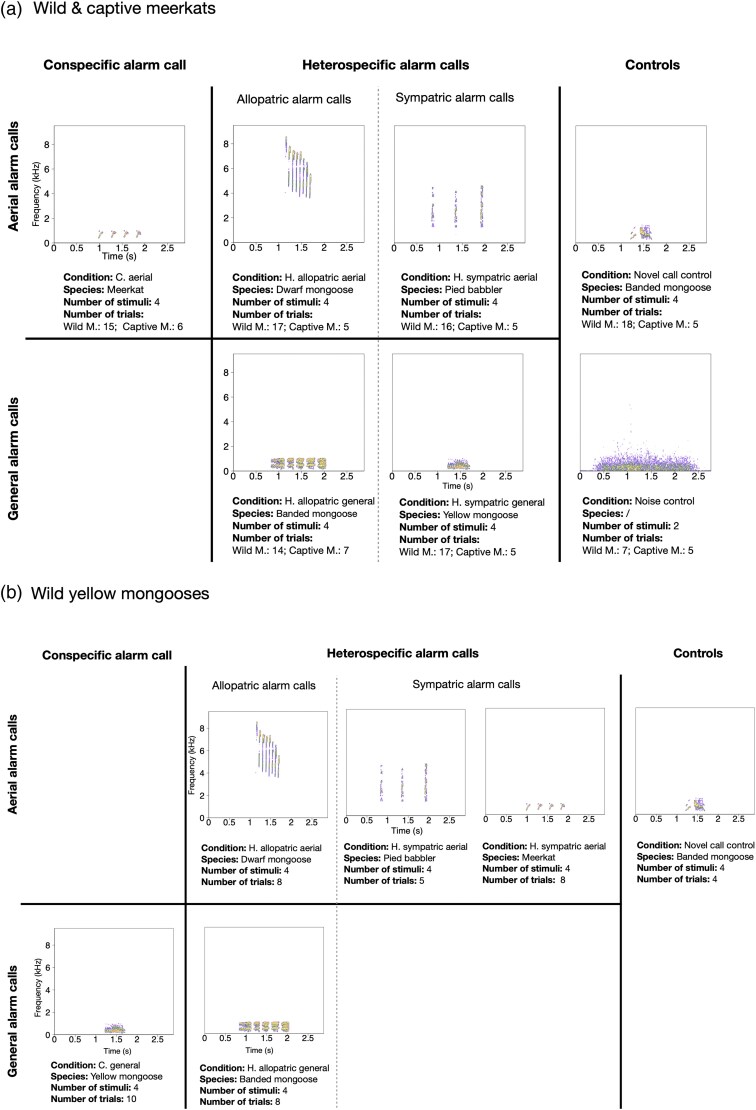
Alarm call conditions tested in the playback experiment: a) wild and captive meerkat experiments; b) wild yellow mongoose experiments. The illustrated calls are examples of each condition, and each condition contained four (two in case of the noise control) representations of the respective type of alarm call. Below each spectrogram, we provide information on the species that produced the alarm call, number of unique stimuli used for each condition and how many trials were conducted.

In our study, we designed a playback experiment to investigate the ability of two sympatric mongoose species in the wild, meerkats and yellow mongooses, to eavesdrop on each other's as well as on the sympatric pied babblers' alarm calls, and also on alarm calls from various heterospecific mongoose species, the dwarf mongooses and banded mongooses. Specifically, we were interested in whether receivers could recognize these stimuli as alarm calls and show appropriate antipredator behavior. Furthermore, we explored whether any such responses were likely to be based on innate perceptual sensitivities to acoustic features common across alarm call repertoires or rather based on learned associations with the calls. To this end, we played back aerial and general alarm calls produced by both sympatric (pied babblers, yellow mongooses) or allopatric (dwarf mongooses, banded mongooses) species (Fig. 1) to wild meerkats. To probe the possibility of learned alarm call associations, we replicated the experiment with captive meerkats. Here, we aimed to investigate if their responses were similar to those of wild meerkats, even though they had not experienced any of the heterospecific alarm calls nor any predation themselves. Finally, we played back both “aerial” and “general” heterospecific alarm calls to explore whether these functional differences in alarm calls are also salient to eavesdroppers. We examined meerkats and yellow mongooses because they are phylogenetically close to one another (Patou et al. 2009), and share the same home range and predation threats, but differ in their alarm call repertoires (Manser et al. 2014).

The data generated by this experiment was used to test several hypotheses. In Hypothesis 1, we explored whether meerkats and/or yellow mongooses can eavesdrop on heterospecific alarm calls. We predicted that if a species is capable of this, heterospecific alarm calls should elicit a similar behavioral response to conspecific alarm calls. In Hypothesis 2, we tested the extent to which observed eavesdropping was likely to be a result of learned associations between the calls used for playback stimuli and a predator encounter, rather than innate perceptual sensitivities to the acoustic features generally found in alarm calls across species. We reasoned that if learning plays a role, (a) the subjects would be most likely to engage in predator avoidance behaviors in response to heterospecific sympatric species (ie species with whom they have experience), relative to heterospecific allopatric species (ie species with whom they have no experience). Furthermore, captive meerkats, who have had zero exposure to any of the playback species, would not differ in their behavioral response between sympatric or allopatric heterospecific playbacks (as they have no experience with either), and would have a weaker response to sympatric species than their wild counterparts. On the contrary, if eavesdropping is based on innate perceptual sensitivities to the common acoustic features of alarm calls found across species, we predicted that our subjects, including the captive meerkats, would engage in predator avoidance behavior in response to all alarm playback stimuli, regardless of source. In Hypothesis 3, we examined whether eavesdroppers were able to infer not only the presence of a predator from a heterospecific alarm call, but also the category of threat referred to by the call. If this were the case, we expected to observe predator avoidance behavior consistent with the playback referent (eg scanning the air in response to aerial alarm calls, but not in response to general alarm calls).

## Materials and methods

### Study populations

#### Wild habituated meerkats

We conducted the experiments with a meerkat population in their natural habitat between August and September 2022. The experiments involved eight wild, but habituated meerkat groups studied within the long-term Kalahari Meerkat Project, located at the Kalahari Research Center (26°58′S, 21°49′E). All individuals were dye-marked for easy individual recognition (Jordan et al. 2007). Two adult focal individuals, a male and a female, were selected from each group, with group sizes ranging from seven to 21 individuals. If the focal individual was not with the group, or it left the group during the experiment, a different focal individual was selected of the same age category and sex. This resulted in a total sample of 20 focal individuals (male = 11, female = 9). We conducted a total of 104 playback trials, distributed within an average of 5.25 sessions per group (minimum = 4, maximum = 6) (see Table S1 for more details). To prevent habituation of the meerkats to the stimuli, each group was exposed to a maximum of one test session per week, and with a maximum of 3 trials per session per group. The experiment was conducted at least 15 meters from the sleeping burrow and 5 meters from a bolthole, which are holes in the ground that meerkats and yellow mongooses use as refuge.

#### Wild habituated yellow mongooses

The yellow mongoose experiment took place at the same study site as the experiments on the wild meerkats (Kalahari Research Center) between September 2022 and April 2023. As with the wild meerkats, the yellow mongooses were dye-marked for identification and habituated to human presence. A total of ten (males = 5, females = 5) yellow mongooses from six (one individual changed groups during the experiment and was then tested in the other) groups were tested (see Table S1). Due to the reason that yellow mongoose groups tend to be small and not all individuals were habituated enough to participate in the experiment, only a single individual was tested from some groups. The experiment was only carried out while subjects were foraging and when the focal individual was at least 10 meters away from the sleeping burrow. Since yellow mongooses predominantly forage solitarily, the experiment was conducted when the focal individual was foraging by itself. There was one exception to this, where a second individual was also present within close distance (∼5 m). The responses to the playback in this trial were compared with those in other trials, showing the presence of the nonfocal individual seemed not to affect any of the response variables of the focal. In total, 43 playback trials were conducted. We conducted an average of 3 sessions per individual (minimum = 1, maximum = 4) (See Table S1). As with the meerkats, we only carried out one session a week to avoid habituation to the playback stimuli, with a maximum of two trials per individual per session, whereby a session lasted ∼1 h each. The number of possible trials per session was reduced in comparison to the wild meerkats, because yellow mongooses spent less time foraging during the day.

#### Captive meerkats

The playback experiments with captive meerkats were conducted from the July 2023 to October 2023, testing ten meerkats (males = 4, females = 6) from four groups at various enclosures in Switzerland (University of Zurich Irchel, Zoo Zurich, Knies Kinderzoo and Walter Zoo). Since it was not feasible to dye-mark the meerkats at the zoos, we selected two adult individuals per group that could be easily recognized by physical features (eg deformed tail, scars, distinct fur colors). To ensure the focal individuals were foraging during the playback trial, we performed the experiments 5 to 10 min after the meerkats had been fed. To elicit foraging behavior most similar to that of wild meerkats, food items were spread out as far as possible within the enclosure. Furthermore, the experiment was conducted at least 10 meters from the sleeping burrow and 2 to 3 meters from a bolthole. In comparison to the wild meerkats and yellow mongooses, these distances to the sleeping burrow or boltholes had to be reduced, because of the spatial constraints of the enclosures. Due to a seasonal temperature drop, playback experiments with the Walter Zoo meerkat group was stopped before the full set of trials could be completed. This affected 5 test sessions (10 playback trials), which reduced the sample sizes for the following conditions: Heterospecific Allopatric Aerial Alarm (2 trials less), Heterospecific Allopatric General (1 trial less), Heterospecific Sympatric Aerial (2 trials less), Heterospecific Sympatric General (1 trial less), Novel Call Control (2 trials less), Noise Control (2 trials less). This meant we were only able to conduct two sessions with two successful trials in total (Table S1). Across all locations, we conducted a total of 37 playback trials, with a maximum of 2 trials per individual per session. Despite the absence of predation, it has previously been shown that captive meerkats have the same alarm call repertoire and produce the same alarm call types in contexts that resemble those in the wild (Hollén and Manser 2007).

### Conditions and stimuli

To determine whether meerkats (wild/captive) respond differently to aerial and general alarm calls of allopatric and sympatric species, we constructed seven playback conditions (Fig. 1a). The alarm calls presented varied on two levels: alarm call types (aerial alarm calls vs. general alarm calls) and home range (sympatric species vs. allopatric species) (Fig. 1a). In the wild and captive meerkat experiments (Fig. 1a), allopatric aerial alarm calls were used from dwarf mongooses (dwarf mongoose “type 1” alarm calls, (Collier et al. 2017), allopatric general alarm calls from banded mongooses (“worried” calls, Jansen 2013), sympatric aerial alarm calls from pied babblers and sympatric general alarm calls from yellow mongooses (“rolling alarm”, Le Roux et al. 2009). Dwarf mongoose, banded mongoose and yellow mongoose alarm calls were selected due to these species being closely related to meerkats but having different alarm call repertoires. There was, however, no sympatric mongoose species available that produces an aerial alarm call. For this reason, the alarm call of a local avian species (pied babbler) was selected instead. For the experiments with meerkats, conspecific aerial alarm calls and two control conditions (novel call control and noise control) were also played back to establish a reference of the natural response of meerkats to their conspecific aerial alarm calls (Fig. 1a) to which responses to heterospecific calls could be compared. The novel call control was used to test the response to novel nonalarm calls, whereby we used banded mongoose “lead” calls, which are typically produced when individuals try to induce movement; (Furrer 2010; Jansen 2013). Specifically, this playback condition was designed to test whether meerkats respond to any novel call as though it were a threat, or if they instead differ in their responses between novel alarm stimuli and nonalarm stimuli (Fig. 1). Additionally, the noise control (prerecorded background noise from the local habitat) was played back to control for the possibility that individuals simply responded to any type of sound played back to them (Fig. 1). Each condition contained four stimuli of different recordings of the same call type, originating from different alarm events and individuals. When repeating the experiment with yellow mongooses, we slightly adjusted the playback conditions. In the wild yellow mongoose experiment (Fig. 1b), the conspecific alarm calls were recordings from yellow mongooses, allopatric aerial alarm calls from dwarf mongooses and sympatric aerial alarm calls from pied babblers and meerkats. There was, however, no sympatric general alarm call condition. Lastly, we only played back the novel call control, but not a noise control condition, because we were limited in the number of trials we were able to carry out with the yellow mongooses.

The playback stimuli were created with Adobe Audition (version 22.1.1.23) and PRAAT (version 6.2.01) (Boersma and Weenik 2024). Each of the playback stimuli were constructed from a unique recording of a vocalizing individual, so that no two stimuli were drawn from the same recording. Audio files with good audio quality were selected (low background noise, no clipping). If files stemmed from a longer sequence, we selected the vocalizations from the beginning of the sequence. The selected calls ranged from one to 10 acoustic elements, separated by short inter-element-intervals (see Table S2 for details). All stimuli were amplitude equalized to 70 dB SPL (60 dB SPL at 1 m from the speaker) using the PRAAT function “Scale intensity”, which does not apply frequency filtering or weighing. The amplitude at 1 m from the speaker was measured using a sound level meter (Volcraft, SL-100, measure range 30 to 130 Db A/C Precision ± 22 Db). The stimuli varied in length from 3.05 s to 4.71 s, consisting of a bout of 1 to 5 individual elements (Table S2). To minimize variation in the background noise between stimuli, a 500 ms segment of background noise was artificially inserted directly before and after the stimuli. The same segment of background noise was used for all stimuli to maintain consistency. Furthermore, all stimuli contained a fade in/fade out (∼400 ms) to eliminate a sudden onset of background noise.

Prior to the experiment, stimuli were randomly assigned to focal individuals and sessions. The selected groups were randomly assigned to two stimuli of each condition by using a random number generator until all stimuli were assigned to two groups. Then again by using a random number generator, the order of presentation was determined. Per session, a maximum of 3 trials in wild meerkats, 2 in captive meerkats, and yellow mongooses were planned. To avoid overexposure of the group to the same condition, we ensured that each condition was only played back once per session. Additionally, we made sure that the two selected individuals from the same group were assigned a different stimulus of the same condition. If a trial was not successful (eg the playback experiment overlapped with an alarm event), we repeated the trial with the same individual (if available) and stimulus in a different session.

### Playback procedure

For the wild meerkats, a test session was either carried out in the morning (∼07:15 to 12:30) or in the evening (∼15:00 to 18:00) and typically lasted two and a half hours. The sessions with the yellow mongooses were also either carried out in the morning (∼05:45 to 10:00) or in the evening (∼17:00 to 19:30) typically lasted for one and a half hours. To avoid biases resulting from whether the playback was carried out in the morning or in the evening, we ensured that each group had about an equal number of sessions in the morning and in the evening over the complete experimental period. We also made sure that the stimuli belonging to the same condition were balanced across morning and evening session (eg Conspecific Alarm Stimulus 1—Morning Week 1; Conspecific Alarm Stimulus 2—Evening Week 3). We carried out test sessions with captive meerkats only in the mornings (∼7:30 to 12:30) to avoid peak visiting times at the zoo. A session with the captive meerkats typically lasted about one and a half hours.

To minimize the risk that observed responses were due to human presence, the group (or focal individual in the case of the yellow mongooses) was followed for 30 min before starting the first playback of each session. Between playback trials within a session, the group or individual was followed for 30 min with no interventions to allow them to return to a baseline behavioral and emotional state. In the case of naturally occurring alarm event, eg an individual spotting a predator and running to shelter, we waited an additional 10 min before starting a playback. The focal individual was filmed with a GroPro 8 for one minute before the playback started until at least one minute after the playback, or until the individual was foraging for longer than 10 s. The stimuli were played back with a Zoom Go Pl2 Bluetooth Speaker, placed 2 to 4 meters from the focal individual. When testing the wild meerkats, the speaker was mounted on a tripod at 30 to 40 cm height, which is approximately the height of a bipedal vigilant meerkat. A few minutes before the start of a trial the tripod was placed at the correct distance and if possible, disguised behind grass or a bush. The experimenter made sure that the focal individual was not responding to the placing of the speaker and was continued to be followed until the start of the playback trial. When testing the yellow mongooses and captive meerkats, the speaker was strapped to the experimenter's leg at 30 to 40 cm height. This change of method was implemented to make it easier for the experimenter to keep sight of the focal individual, because it was more difficult to follow the wild yellow mongooses (who were less habituated than wild meerkats, spend less time foraging on a single foraging patch, and move faster through dense vegetation) and captive meerkats (who were also less habituated and mostly not dye-marked) during the experiment.

### Analysis

#### Video analysis

All behaviors of meerkats and yellow mongooses were coded in the one minute before and after onset of the playback stimulus using the video coding software BORIS (Friard and Gamba 2016). Coding was conducted frame-by-frame (59 fps), and the audio of the video was muted to ensure that the coders did not know the stimulus the focal individual was exposed to. To determine when the playback stimulus was played, we extracted the audio file and set a marker to the start of the playback in Adobe Audition. If the focal individual was already vigilant at the time of the onset of the playback stimulus, the trial was excluded from the analysis (wild meerkats = 7 trials, wild yellow mongooses = 7 trials, captive meerkats = 1 trial). We measured two primary response variables, fleeing and looking up into the sky (Table 1), to test our three hypotheses, as these reflect behaviors expected of meerkats when exposed to their conspecific aerial alarm call. We also quantified four additional variables, freezing, duration of the first gaze event, latency to first response, and latency to relaxation (Table S3), to describe mongoose responses to our playback stimuli across conditions. For the yellow mongooses and the captive meerkats, we omitted the freeze response from the analysis, because neither of these groups showed this behavior. All videos were coded by NF, and to ensure the robustness of the measured response variables, we conducted an inter-observer reliability test where 63% of the videos were coded by a second observer AMW. To test the agreement between the two observers, we calculated Cohen's Kappa for the categorical variables and the interclass correlation scores (ICC) for the continuous variables and achieved an average of 0.76 (scores between 0.75 and 0.9 are generally considered as “good” or “substantial” inter-rater reliability, (McHugh 2012; Koo and Li 2016).

**Table 1:** Definition of behavioral response variables quantified in the playback experiments to test hypothesis.

Behavior	Type of data	Description
Flee	Categorical	Fleeing as an immediate response to the playback stimulus (y/n) Only running from the sound source within the first 10 s after the onset of the playback were counted as an immediate flee response to the playback. Running at a later point was rare, and often linked to other behaviors (eg renovating sleeping burrows or boltholes)
Look up	Categorical	Looking up into the sky (y/n) While looking up, the head was tilted upward to a more than parallel angle from the ground.

See Table S3 for details on additional response variables, which were used to assess general behavioral responses: freezing, duration of first gaze, time until first response and time until relaxation.

### Statistical analysis

We evaluated whether wild and captive meerkats as well as wild yellow mongooses responded differently to heterospecific aerial and general alarm call types of allopatric and sympatric species by fitting Bayesian models. For the categorical response variables (flee, look up, freeze), we fitted one Bayesian Bernoulli model for each response variable with playback condition (conspecific alarm call, sympatric aerial alarm call, sympatric general alarm call, allopatric aerial alarm call, allopatric general alarm call, novel call control, noise control) and study population (wild meerkat, captive meerkat, wild yellow mongooses) were added as interacting categorical fixed effects. For the continuous response variables (duration until the first response, duration of the first gaze event and relax duration), we fitted a Bayesian hurdle log-normal regression for each response variable again with playback conditions, populations and their interaction as fixed effects. The model estimated both the probability of a nonzero duration, and conditional of it occurring, the length of that duration. For all models, random intercepts for Group ID and Individual ID were included to account for hierarchical structure and repeated measures. The model formula was: Response Variable ∼ Playback Condition × Study Population + (1|Individual ID) + (1| Group ID). Posterior predictions were then used to estimate marginal effects for each call type-population combination, from which we extracted and summarized posterior probability estimates and their 95% credible interval.

To evaluate our hypotheses, we focused our further analyses on the “flee” and “looking up into the sky” behaviors, because they reflected the behavior we expected meerkats to show when exposed to their conspecific aerial alarm call and gave us therefore clear expectations of how responses would differ depending on call types. The posterior draws were extracted from these two models, which were then used to estimate contrasts between playback conditions and study populations, along with their corresponding 95% credible intervals. Contrasts were carried out for each level of the fixed effect of playback type to determine whether they elicited robust differences in the outcome measure, indicated by instances where the 95% credible interval of the posterior estimate did not cross zero. First, we compared the contrasts between the noise control and all other conditions for each study population, and the contrasts between the novel call control and all heterospecific alarm calls. For hypothesis 1, we compared the contrasts between the responses to conspecific alarm calls and heterospecific alarm calls. For hypotheses 2, we compared heterospecific aerial alarm calls of sympatric and allopatric species and heterospecific general alarm calls of sympatric and allopatric species. Additionally, we compared the responses of wild and captive meerkats to con- and heterospecific alarm calls. Lastly, for hypothesis 3, we compared the responses to heterospecific aerial and general alarm calls.

All models were implemented in R (version, 4.5.2, R Core Team 2025) using the package “brms” (version 2.23.0) (Bürkner 2017), contrasts were calculated using the package “stats” (version 4.5.2). The raw data and contrasts were illustrated using the R packages “ggplot2” (version 3.5.0). All data and R scripts are available at the open science framework (https://osf.io/9rjk7/?view_only=60b28f05b872409db2eb93b1d2748d11).

## Results

**Figure 2:**
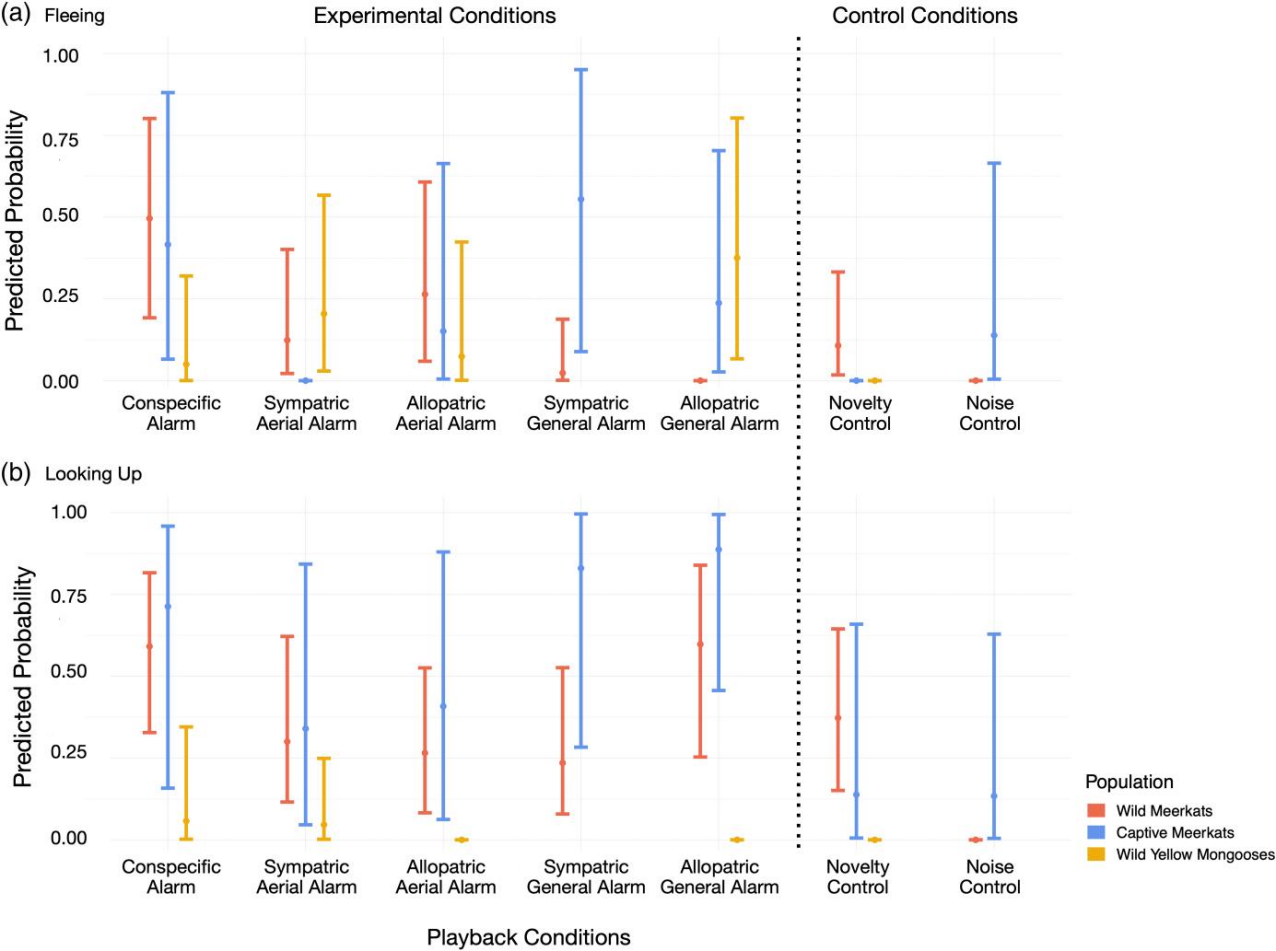
Predicted probability to a) flee and b) look up into the sky as a response to conspecific alarm calls and heterospecific aerial and general alarm calls, produced by either sympatric or allopatric species in wild (red) and captive meerkats (blue) and wild yellow mongooses (yellow). Each playback condition shows the mean (central dot) and the 95% credible interval (edge lines). See Tables S4 and S5 for details and Tables S6–S11 and Figures S1–S4 for additional response variables (freeze, time until first response, duration of first gaze event and time until relaxation).

We first explored whether meerkats showed a different behavioral response to the noise control than to calls produced by animals. We found that in comparison to the noise control, wild meerkats were more likely to flee (except for the allopatric general alarm calls) and look up into the sky as a response to all other stimuli (Fig. 2, Tables S4, S5, S10, and S11). Furthermore, captive meerkats were more likely to look up into the sky when exposed to heterospecific general alarm calls than the noise control, but they were not more likely to look up into the sky when exposed to heterospecific aerial alarm calls, conspecific alarm calls and the novel call control than the noise control. There was no difference in the probability to flee between the noise control and most other conditions in captive meerkats (Fig. 2, Tables S4, S5, S10, and S11). However, captive meerkats were more likely to flee as a response to the noise control when compared with the novel call control and sympatric aerial alarm calls control. Given the strength of response by captive meerkats to the noise control, it is plausible that responses to playback stimuli can be attributed to some element of the experimental setup rather than genuine responses to vocal stimuli. Specifically, the amplitude of the noise control was adjusted in some instances, as the original stimulus was masked by background noise in the zoo environment. Such adjustments may have inadvertently influenced the animals' responses. The interpretation of the corresponding data should therefore be carried out with caution.

When we investigated whether meerkats and yellow mongooses respond differently to heterospecific alarm calls than to the novel call control, we found differences among the populations in regard to fleeing and looking up into the sky. Wild meerkats were not more likely to flee or look up into the sky as a response to heterospecific alarm calls relative to the novel call control (Fig. 2, Tables S4, S5, S12, and S13). Captive meerkats, on the contrary, were more likely to flee as a response to heterospecific alarm calls in comparison to the novel call control (Fig. 2, Tables S5 and S12). Furthermore, captive meerkats were more likely to look up into the sky as a response to heterospecific general alarm calls, but not heterospecific aerial alarm calls, relative to the novel call control (Fig. 2, Tables S4 and S13). In terms of fleeing responses, yellow mongooses were more likely to react to conspecific and heterospecific alarm calls in comparison to the novel call control (Fig. 2, Tables S5 and S12). Considering looking up into the sky, they were more likely to react to their conspecific general alarm call and the aerial alarm calls of sympatric species compared with novel call controls (Fig. 2, Tables S5 and S13) but showed no difference in response between allopatric alarm calls and the novel call control (Fig. 2, Tables S5 and S13).

*Hypothesis 1: Meerkats and yellow mongooses can eavesdrop on the alarm calls of heterospecific species.*

**Figure 3:**
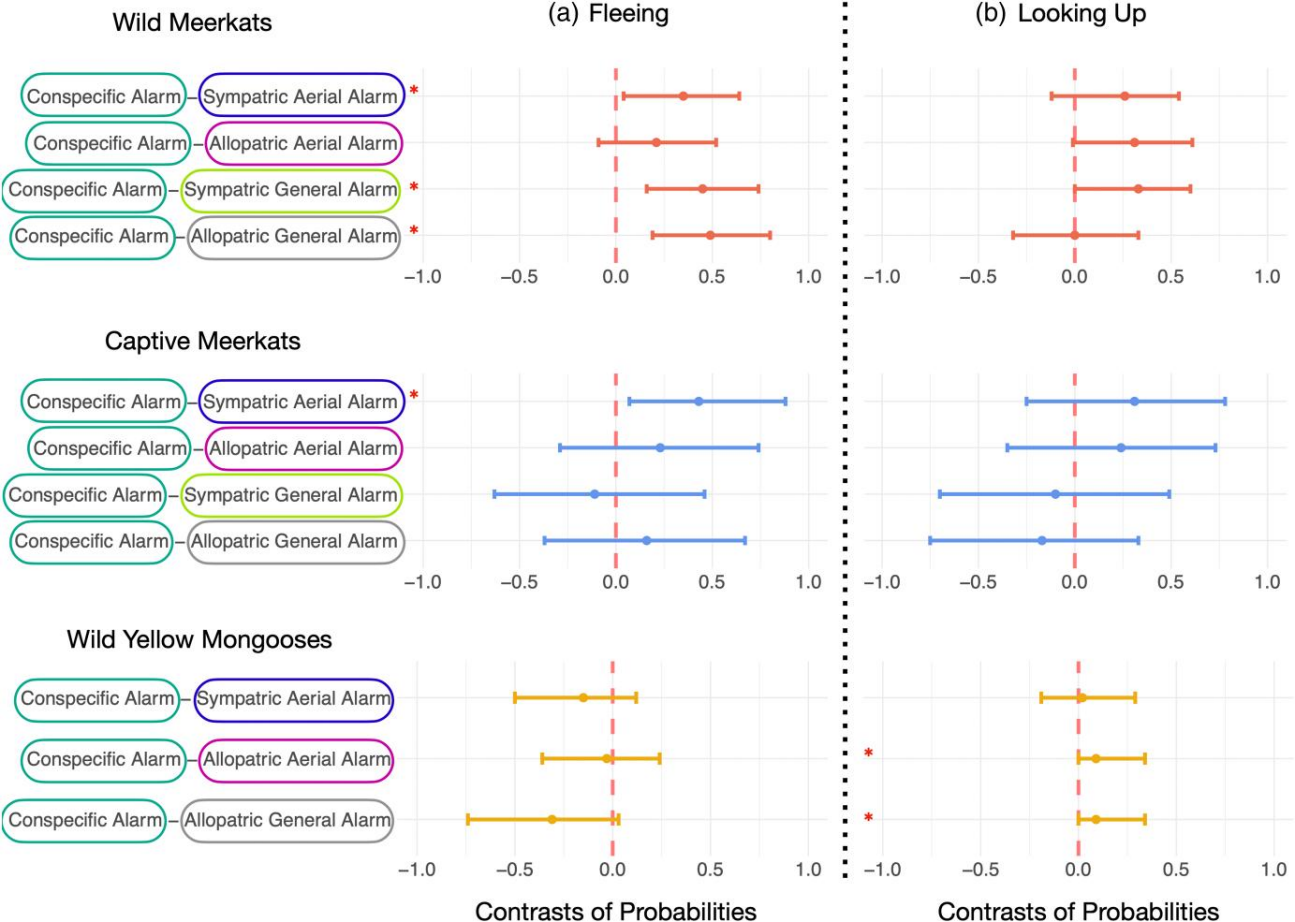
Contrasts between conspecific and heterospecific alarm calls for each species. This figure corresponds to Hypothesis 1: Meerkats and yellow mongooses can eavesdrop on the alarm calls of heterospecific species. Shows: a) fleeing and b) looking up as a response to conspecific alarm calls and heterospecific alarm calls in wild and captive meerkats and wild yellow mongooses. The vertical red lines illustrate the mean estimate, horizontal lines represent the 95% credible interval of the estimate. Horizontal lines that do not cross the vertical red line indicate a robust difference between two conditions. Robust differences are also marked with an asterisk. See Fig. 2 for absolute responses for each condition and Tables S14 and S15 for details.

When testing whether subjects are capable of eavesdropping on heterospecific alarm calls and respond to these in a behaviorally similar way as to conspecific alarm calls, we found mixed evidence. In comparison to conspecific alarm calls, wild meerkats were less likely to respond by fleeing as a response to most heterospecific alarm calls (conditions: sympatric aerial alarm call, sympatric general alarm call, allopatric general alarm call) (Fig. 2, Fig. 3a, Table S14). However, the probability to flee did not robustly differ from conspecific alarm calls when responding to heterospecific aerial alarm calls of an allopatric species (Figs. 2 and 3a, Table S14). There was no difference in the probability to look up into the sky between conspecific and any heterospecific alarm calls (conditions: sympatric aerial alarm call, allopatric aerial alarm call, sympatric general alarm call, allopatric general alarm call) (Figs. 2 and 3b, Table S15). For captive meerkats, the probability to flee (Figs. 2 and 3a, Table S14) and look up (Figs. 2 and 3b, Table S15) did not robustly differ between most playback conditions of conspecific and heterospecific alarm calls, with the exception of aerial alarm calls produced by a species sympatric to wild meerkats, which were reliably less likely to elicit fleeing behavior than conspecific calls (Figs. 2 and 3a, Table S14). Wild yellow mongooses did not differ in the probability to flee between conspecific general alarm calls and heterospecific alarm calls (conditions: sympatric aerial alarm call, allopatric aerial alarm call, allopatric general alarm call) (Figs. 2 and 3a, Table S14). Compared with conspecific alarm calls, yellow mongooses were less likely to look up in the sky when exposed to heterospecific allopatric alarm calls (conditions: allopatric aerial alarm, allopatric general alarm), but not heterospecific sympatric aerial alarm calls (Figs. 2 and 3b, Table S15).

*Hypothesis 2: Eavesdropping is a product of learned associations with heterospecific alarm calls.*

**Figure 4:**
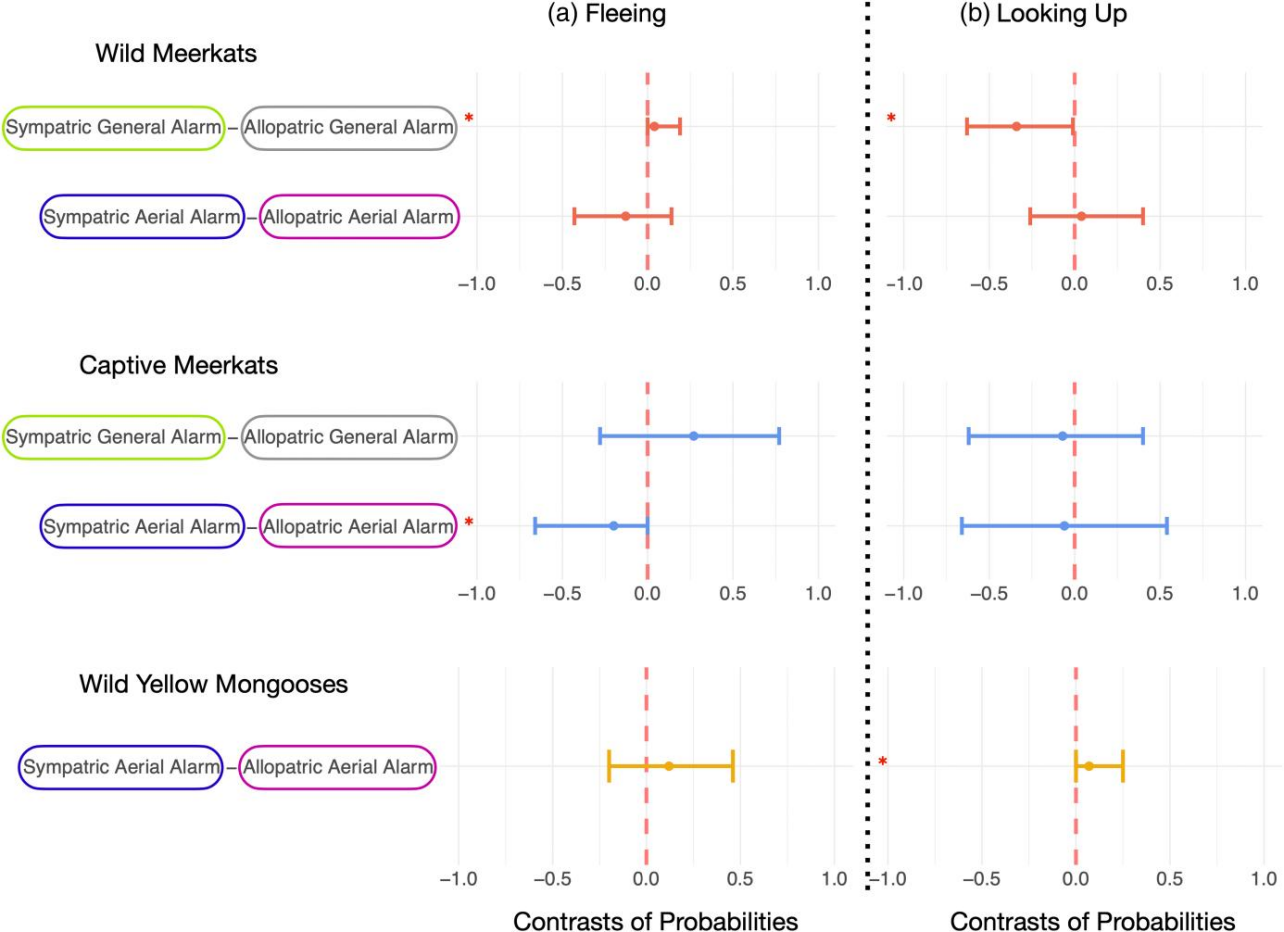
Contrasts between heterospecific alarm calls produced by either sympatric or allopatric species a) fleeing and b) looking up as a response to heterospecific aerial alarm calls and heterospecific general alarm calls produced by either sympatric or allopatric species in wild and captive meerkats and wild yellow mongooses. This figure corresponds to Hypothesis 2: Eavesdropping is a product of learned associations with heterospecific alarm calls. The vertical red lines illustrate the mean estimate; horizontal lines represent the 95% credible interval of the estimate. Horizontal lines that do not cross the vertical red line indicate a robust difference between two conditions. Robust differences are marked with an asterisk. See Fig. 2 for absolute responses for each condition and Tables S16 and S17 for details.

**Figure 5:**
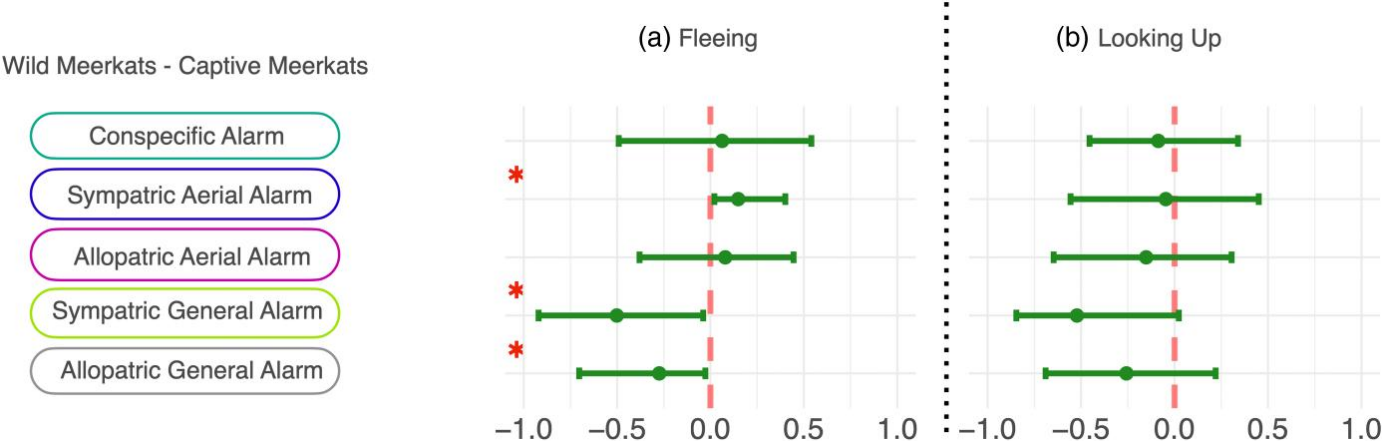
Contrasts between wild and captive meerkats for conspecific and heterospecific alarm calls: a) fleeing and b) looking up as a response to conspecific and heterospecific alarm calls. This figure corresponds to Hypothesis 2: Eavesdropping is a product of learned associations with heterospecific alarm calls. The vertical red lines illustrate the mean estimate; horizontal lines represent the 95% credible interval of the estimate. Horizontal lines that do not cross the vertical red line indicate a robust difference between two species. Robust differences are also marked with an asterisk. See Fig. 2 for absolute responses for each condition and Tables S18 and S19 for details.

When testing whether eavesdropping is a product of learned associations, we found that, compared with allopatric general alarm calls, wild meerkats were more likely to flee and less likely to look up in the sky as a response general alarm calls of sympatric species (Fig. 2, Fig. 4a, Tables S16 and S17). We did not find this effect for heterospecific aerial alarm calls (Figs. 2 and 4a, Tables S16 and S17). Captive meerkats, on the contrary, were more likely to flee as a response to aerial alarm calls that are allopatric to wild meerkats, compared with aerial alarm calls of species that are sympatric to wild meerkats (Figs. 2 and 4a, Table S16). Wild yellow mongooses were more likely to look up in the sky, but not more likely to flee, when exposed to the aerial alarm calls of sympatric species than allopatric species (Figs. 2 and 4a, Tables S16 and S17). Additionally, wild and captive meerkats did not differ in their probability to flee or look up into the sky as a response to conspecific alarm calls and allopatric aerial alarm calls (Fig. 2, Fig. 5, Tables S18 and S19). Wild meerkats were robustly more likely to flee as a response to the heterospecific aerial alarm calls of a sympatric species in comparison to captive meerkats (Figs. 2 and 5, Tables S18 and S19). Furthermore, captive meerkats were robustly more likely to flee as a response to heterospecific general alarm calls, both of allopatric and sympatric species, in comparison to wild meerkats (Figs. 2 and 5, Tables S18 and S19).

*Hypothesis 3: Eavesdroppers can infer the category of threat referred to by heterospecific calls.*

**Figure 6:**
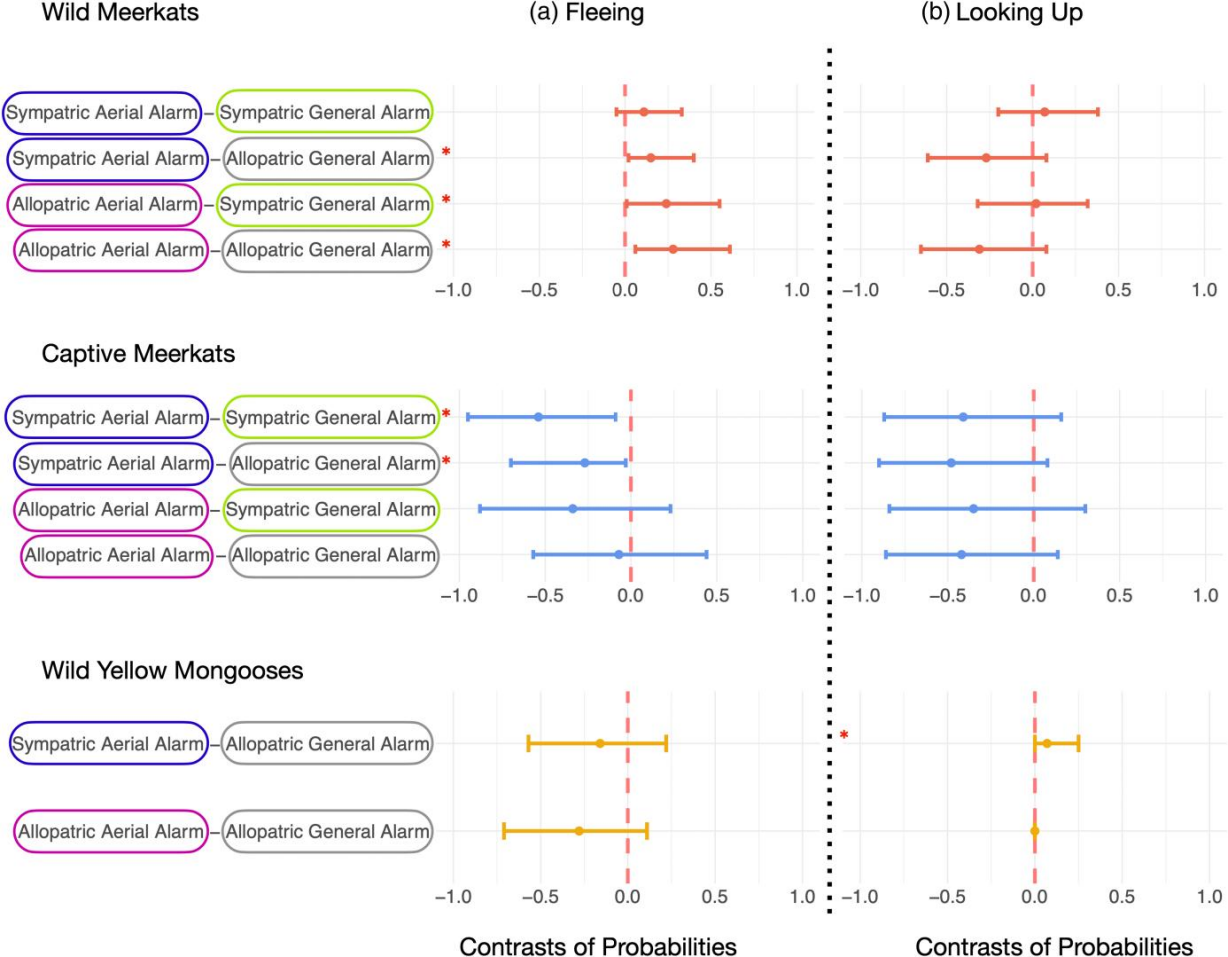
Contrasts between heterospecific aerial and general alarm calls a) fleeing and b) looking up as a response to heterospecific aerial alarm calls and heterospecific general alarm calls in wild and captive meerkats and wild yellow mongooses. This figure corresponds to hypothesis 3: Eavesdroppers can infer the threat referred to by heterospecific calls. The vertical red lines illustrate the mean estimate, horizontal lines represent the 95% credible interval of the estimate. Horizontal lines that do not cross the vertical red line indicate a robust difference between two conditions. Robust differences are also marked with an asterisk. See Fig. 2 for absolute responses for each condition and Tables S20 and S21 for details.

When testing whether eavesdroppers would be more likely to look up into the sky following playbacks of heterospecific aerial alarm calls relative to heterospecific general alarm calls (indicating their ability to infer the type of threat), we found that wild meerkats were more likely to flee as a response to heterospecific aerial alarm calls, but they did not differ in their probability to scan the sky in comparison to heterospecific general alarm calls (Fig. 2, Fig. 6, Tables S20 and S21). For captive meerkats, we found that they were more likely to flee as a response to heterospecific general alarm calls, compared with the aerial alarm calls of a sympatric, but not allopatric species (Figs. 2 and 6, Tables S20 and S21). Wild yellow mongooses were more likely to scan the sky when exposed to the aerial alarm calls of sympatric, but not allopatric, species (Figs. 2 and 6, Tables S20 and S21).

## Discussion

In this study, we aimed to identify the mechanisms underlying heterospecific eavesdropping behavior in mongooses. We did so by testing whether wild and captive meerkats, as well as wild yellow mongooses, responded to heterospecific aerial and general alarm calls in functionally appropriate ways. By exposing these three populations to playbacks of alarm calls from sympatric and allopatric species, we explored three hypotheses: (1) mongooses can eavesdrop on heterospecific alarm calls; (2) eavesdropping is a product of learned associations and/or innate sensitivities to the acoustic features of alarm calls; and (3) eavesdroppers can infer the category of threat referred to by heterospecific alarm calls. In support of the first hypothesis, we found evidence that all three study populations were capable of eavesdropping in some capacity, as they each responded to some heterospecific alarm calls in a similar way to their conspecific alarm calls (Fig. 3). For hypothesis two, we found some evidence that eavesdropping was a learned behavior for wild meerkats. Specifically, relative to captive meerkats, wild meerkats were more likely to flee as a response to heterospecific sympatric aerial alarm calls (Fig. 5), and wild meerkats were more likely to flee as a response to heterospecific general alarm calls from sympatric compared with allopatric species (Fig. 4). Furthermore, yellow mongooses were more likely to look up as a response to alarm calls of familiar, sympatric species as opposed to alarm calls from unfamiliar, allopatric species (Fig. 4). Finally, by testing hypothesis three, we found that only wild meerkats responded in a functionally appropriate way to aerial heterospecific alarm calls (Fig. 3). Taken together, these findings suggest that both learning and innate perceptual sensitivities may play a role in determining the response to heterospecific alarm calls.

Although we found evidence of a learned basis for heterospecific eavesdropping, we argue that mongooses likely have not formed an explicit association to each heterospecific alarm call type. This is indicated by the fact that wild meerkats also showed appropriate antipredation behavior towards allopatric heterospecific aerial alarm calls. Therefore, a possible explanation for the ability of wild meerkats to effectively eavesdrop on allopatric aerial alarm calls could be that they form associations with specific acoustic features of familiar heterospecific alarm calls and generalize these to novel, allopatric calls. Generalization is a learning mechanism that enables responses to novel stimuli that share relevant features with previously experienced stimuli (ten Cate and Rowe 2007). This process reduces the need to learn the details of every new stimulus and often arises in contexts where individuals must discriminate between similar environmental stimuli (Ghirlanda and Enquist 2003; ten Cate and Rowe 2007). For instance, it could be the case that wild meerkats learned a general association between high-pitched, tonal calls and aerial threats. A plausible source of this association is that the aerial alarm calls of avian species are often tonal signals with a high fundamental frequency (Marler 1955; Marler 1956; Magrath et al. 2015a, 2015b). In fact, several such species share their home range with both wild meerkats and yellow mongooses, including Cape starlings (*Lamprotornis nitens*), fork-tailed drongos (*Dicrurus adsimilis*), and ant-eating chats (*Myrmecocichla formicivora*). Generalizing from the acoustic properties of these alarm calls could then lead meerkats to behave similarly to calls that are acoustically similar to one another, even if they have no direct prior experience of them (Magrath et al. 2015a, 2015b). This mechanism could also explain why wild meerkats showed a flee and looking up response to the novel call control playback (a novel but nonalarm banded mongoose call): ie that the stimuli used for this contains acoustic features often found in alarm calls, which the meerkats have a generalized association with. While captive meerkats share the same innate perceptual system as their wild counterparts, and are therefore able to acoustically discriminate between certain heterospecific alarm calls (Fig. 1, Fig. 2), their lack of ecologically relevant experiences, ie predation and their natural soundscape, seems to constrain their ability to respond appropriately.

The ability to generalize associations between known sounds to novel sounds based on common acoustic features is likely to be adaptive, as the potential cost of ignoring alarm calls of heterospecifics is greater than accidentally showing an alarm response to a nonalarm call (Magrath et al. 2015a, 2015b). However, we do not yet understand which species meerkats and yellow mongooses rely on for predator information, or which specific acoustic features they may be attuned to. Identifying these factors, and how they affect behavioral responses to novel sounds, could allow us to better understand the mechanisms involved in the responses to heterospecific alarm calls. One way to achieve this would be to acoustically manipulate playback stimuli to systematically explore the acoustic features meerkats are attuned to. For example, by varying synthetic calls in key acoustic features (peak frequency, frequency modulation rate, or both), previous researchers (Fallow et al. 2013) showed that fairy wrens (*Malurus cyaneus*) flee more often as a response to playbacks that had a more similar peak frequency to their conspecific alarm calls, even if calls varied in other acoustic features. A similar approach could be taken with meerkats by artificially manipulating the acoustic properties of conspecific and heterospecific alarm calls and playing them back to test if the alarm response of meerkats changes with increasing peak frequencies and other acoustic features (eg entropy, tonality, frequency modulations).

Additionally, how mongooses respond to heterospecific alarm calls may be dependent on how energetically costly a certain antipredation behavior is, and the necessity of differentiating one's response to different predator types based on one's living conditions. We found that wild meerkats looked up into the sky more often than yellow mongooses but did not differentiate their “looking up” behavior between stimuli (Fig. 3). This could be due to the relatively low energetic and time cost of the behavior and the high return of information earned when performed. Additionally, wild meerkats, but not captive ones, differentiated their fleeing behavior depending on whether the alarm call was an aerial or general alarm call (Fig. 6). It is possible that populations who spend a greater proportion of time exposed to predation and further away from shelter have a higher need to differentiate their fleeing responses depending on the type of predator encountered, even if their perception of the acoustic and functional differences between heterospecific alarm calls is unchanged. Therefore, it might be necessary for wild meerkats, which are generally more exposed to predation in comparison to both captive meerkats and wild yellow mongooses, to differentiate their physiologically expensive fleeing responses depending on the type of predator encountered. This need for differentiation between threats may also be reflected in the structure of a species' own alarm call repertoire, such as whether it produces specific calls in response to aerial and/or terrestrial predators. For example, it has been argued that banded mongooses do not produce different alarm calls for aerial and terrestrial predators because they are generally close to shelter throughout the day, and therefore have no need to respond differently depending on the type of threat they are facing (Furrer and Manser Marta 2009a, 2009b).

Ultimately, a more holistic approach may be needed to fully understand the factors that lead to eavesdropping between species in the same home range. For this we will need to take into account how signals are both perceived and learned by receivers, and how external factors in the environment (eg predation pressure, distance to shelter) impact eavesdropping on other species. A constraint possibly underlying the results of the present study is that “sympatric” does not necessarily entail “familiar”: it is possible that the wild subjects of this experiment had never been directly exposed to these heterospecific calls, despite overlapping in their home ranges. Furthermore, yellow mongooses rarely produce alarm calls, and although they share sleeping burrows they do not typically forage close to each other, potentially placing them outside the acoustic range of conspecific calls (VR & MM personal observation). Therefore, future studies would benefit from identifying potential “keystone” species, that produce information (eg highly efficient at detecting threats, highly vocal) and from which other species seek information (Magrath et al. 2015a, 2015b). This could help to identify whether individuals of other species are more likely to form associations to calls of these species, and generalize to novel calls that contain similar acoustic features.

In conclusion, our findings suggest that the eavesdropping behavior of both meerkats and yellow mongooses is likely to be at least partially dependent on learning mechanisms such as forming associations generalizing based on familiar acoustic structures, and is therefore contingent on exposure to both predators and the corresponding heterospecific alarm calls. Moreover, we more tentatively suggest that the ability to infer appropriate referent threat categories from these calls may be driven by the ecological need to do so (or lack thereof). However, innate sensitivities to certain acoustic features are also likely to play a role, and future work in this area would benefit from exploring the interaction of these biological, ecological, and learned factors.

## Supplementary Material

arag031_Supplementary_Data

## Data Availability

Analyses reported in this article can be reproduced using the data provided by Falk et al. (2026).
