# Supplementary material for: Exploring mechanisms underlying heterospecific alarm call responses in meerkats and yellow mongooses
Source: Behav Ecol. 2026 Apr 3;37(3):arag031. doi: 10.1093/beheco/arag031 (PMC13107173; doi:10.1093/beheco/arag031)
Supplement: arag031_Supplementary_Data [file arag031_supplementary_data.docx]

**Supplementary Materials**

**
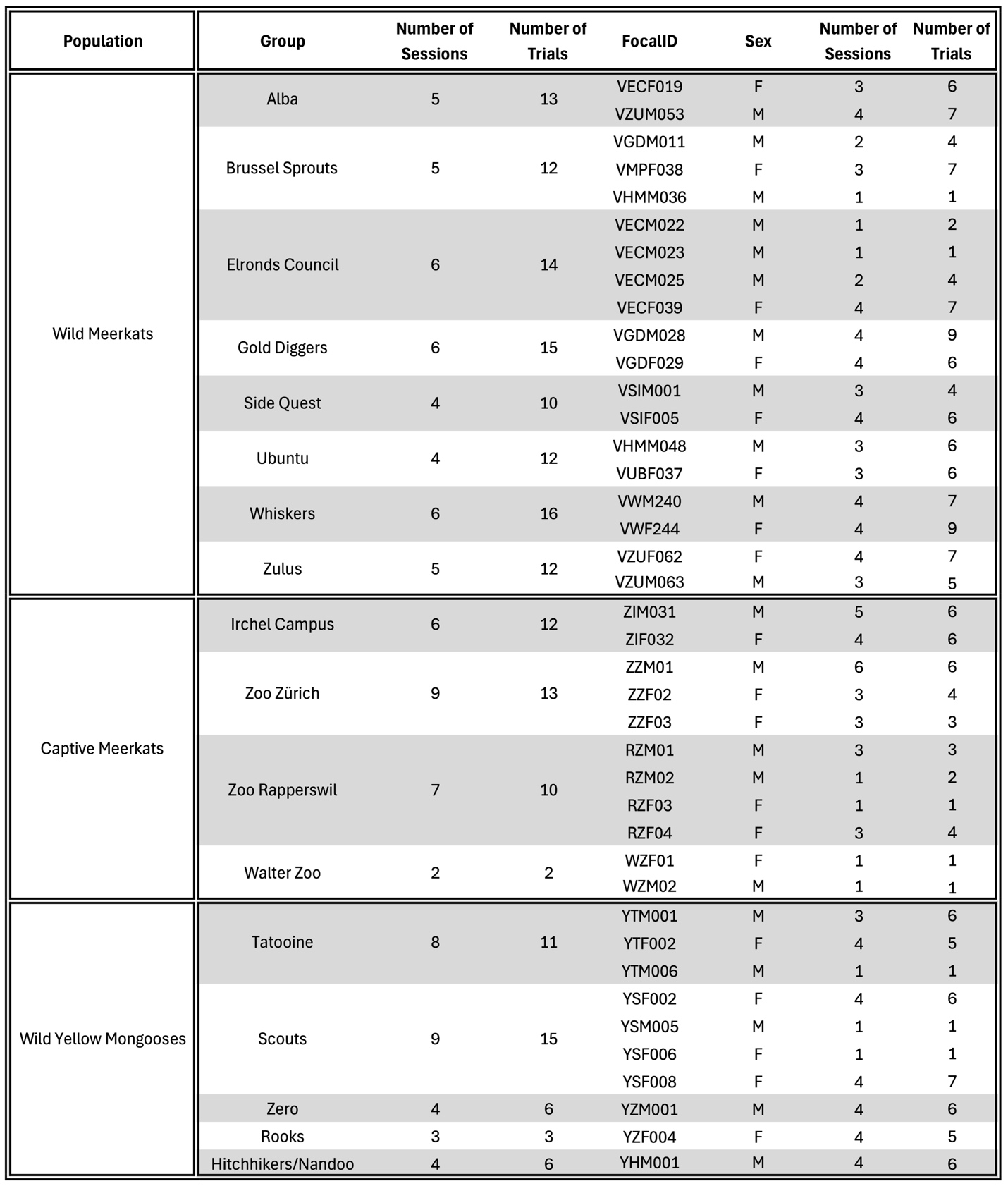
Table S1. List of all groups and individuals used as test subjects.** Number of sessions and trials conducted at each group. Further information is provided on the number of sessions and trials in which the named individuals served as test subjects.

**
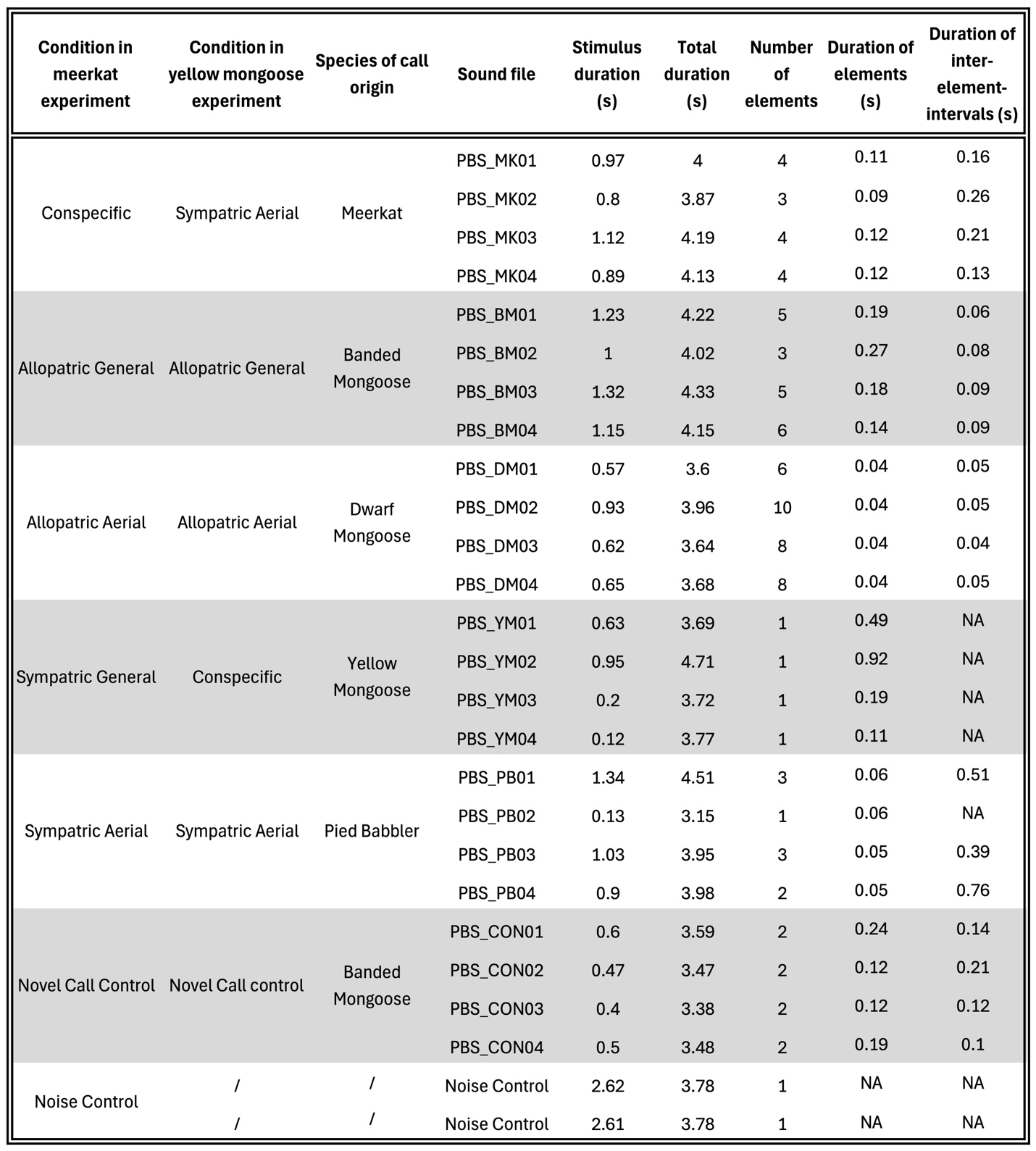
Table S2. List and duration of all stimuli for the various playback conditions.** The Stimulus Duration indicates the duration of the raw stimuli, which only entails the vocalization. The Total Duration indicates the stimuli, including the noise and the silence added before and after each stimulus. Further information is provided on how many elements the vocalization consists of (Number of Elements), the mean duration of these elements and the mean duration of the pauses between these elements (Duration of inter-element intervals).

| **Table S3.** Definition of additional behavioral response variables quantified in the playback experiments.  Behavior Type of data Description | | |
| --- | --- | --- |
| Freeze | categorical | Freezing (y/n).  During freezing behavior, individuals crouched on the ground and did not move for several seconds. |
| Duration of first gaze event | continuous | Duration (in seconds) of the first gaze event of the focal individual after the onset of the playback. The duration of the first gaze event was measured as how long the focal individual gazed in any single direction for the first time after the onset of the playback. Additionally, the gaze duration was measured for each gaze event before and after the playback in the same manner. |
| Time until first response | continuous | Latency (in seconds) until the first change of behaviors of the focal individual after the onset of the playback. |
| Time until relaxation | continuous | Latency (in seconds) until the focal individual forages for a period of at least 5 seconds after the onset of the playback. Because yellow mongooses and captive meerkats hardly foraged for longer than five seconds in their relaxed state prior to the onset of the playback, time until relaxation was adjusted in the following way: foraging or moving for longer than five seconds (yellow mongooses); or foraging, moving or sunning for longer than five seconds (captive meerkats). |

**Table S4. Predicted probability to flee as a response to aerial or general conspecific alarm calls and heterospecific aerial or general alarm calls, produced by either sympatric or allopatric species in wild and captive meerkats and wild yellow mongooses.** For each condition, the estimated probability and 95% credible intervals are provided.

**
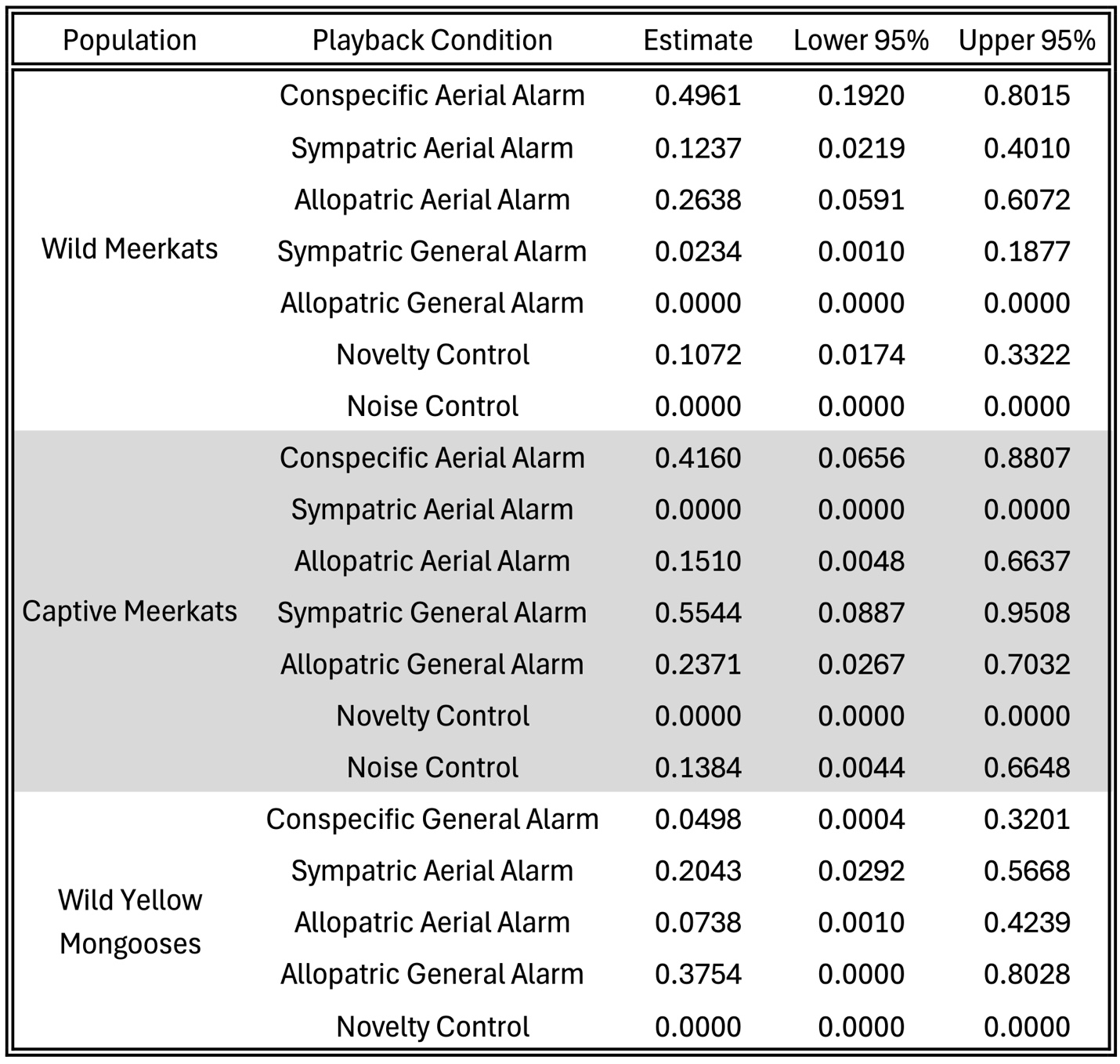
**

**Table S5. Predicted probability to look up into the sky as a response to conspecific aerial or general alarm calls and heterospecific aerial and general alarm calls, produced by either sympatric or allopatric species in wild and captive meerkats and wild yellow mongooses.** For each condition, the estimated probability and 95% credible intervals are provided.

**
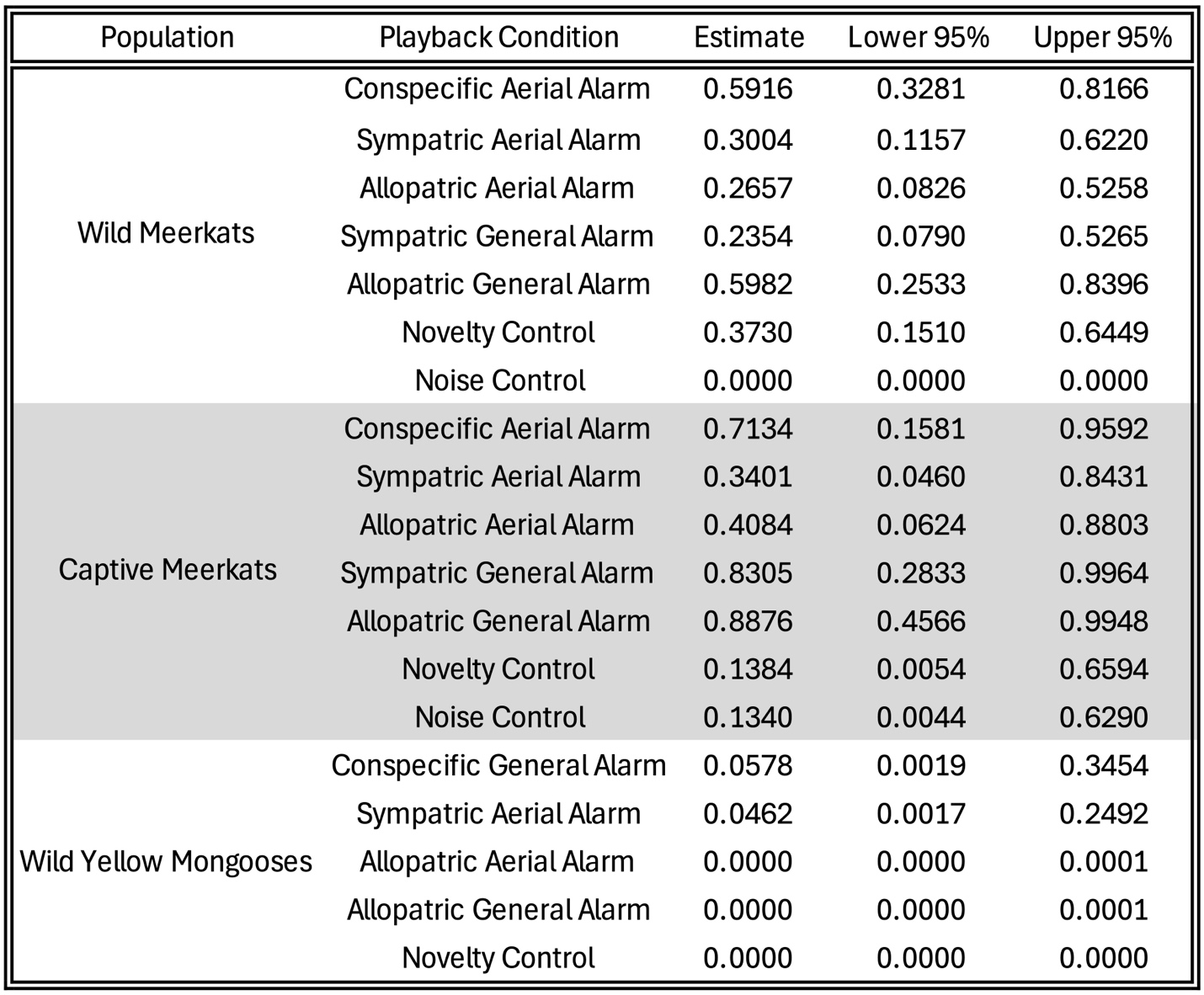
**

**Table S6. Predicted probability to freeze as a response to conspecific aerial or general alarm calls and heterospecific aerial or general alarm calls, produced by either sympatric or allopatric species in wild meerkats.** For each condition, the estimated probability and 95% credible intervals are provided. Note that only wild meerkats showed this behavior. Yellow mongooses and captive meerkats were therefore excluded for this analysis.


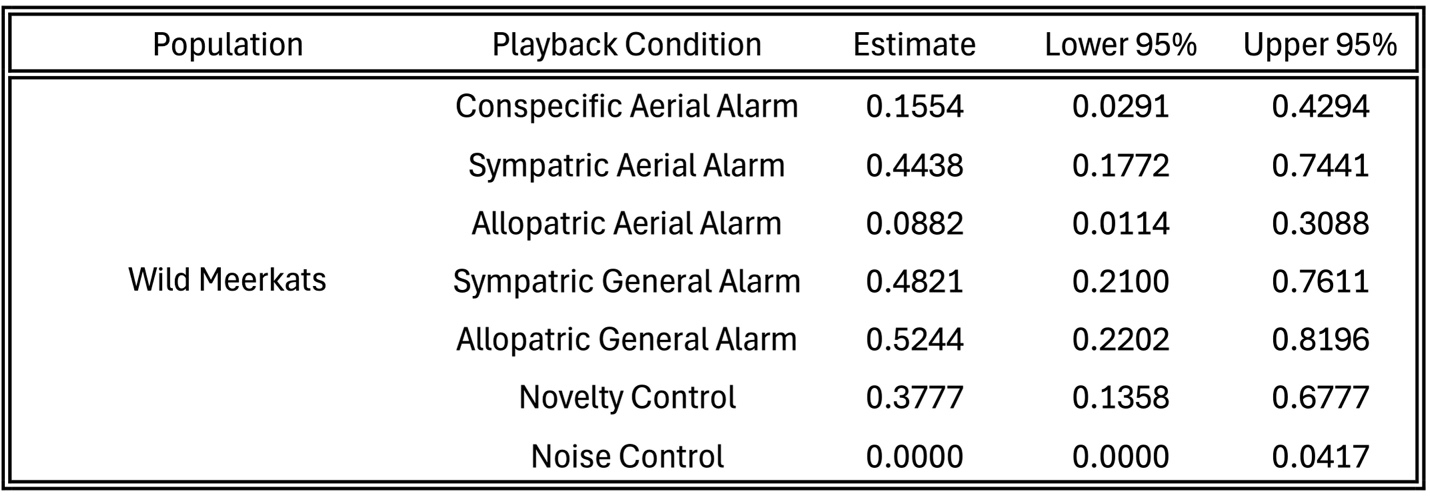


**
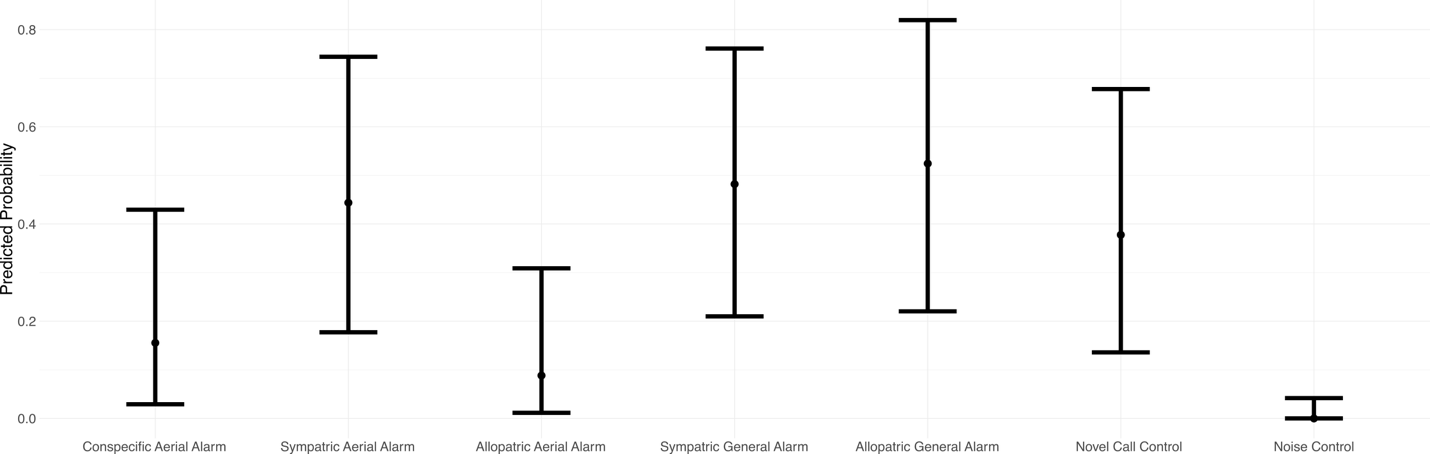
**

**Figure S1.** **Predicted probability to freeze the as a response to conspecific alarm calls and heterospecific aerial and general alarm calls, produced by either sympatric or allopatric species in wild meerkats.** Each playback condition shows the mean (central dot) and the 95% credible interval (edge lines).

**Table S7. Predicted duration until the first response as a response to conspecific alarm calls and heterospecific aerial and general alarm calls, produced by either sympatric or allopatric species in wild and captive meerkats and wild yellow mongooses.** For each condition, the estimated probability and 95% credible intervals are provided.

**
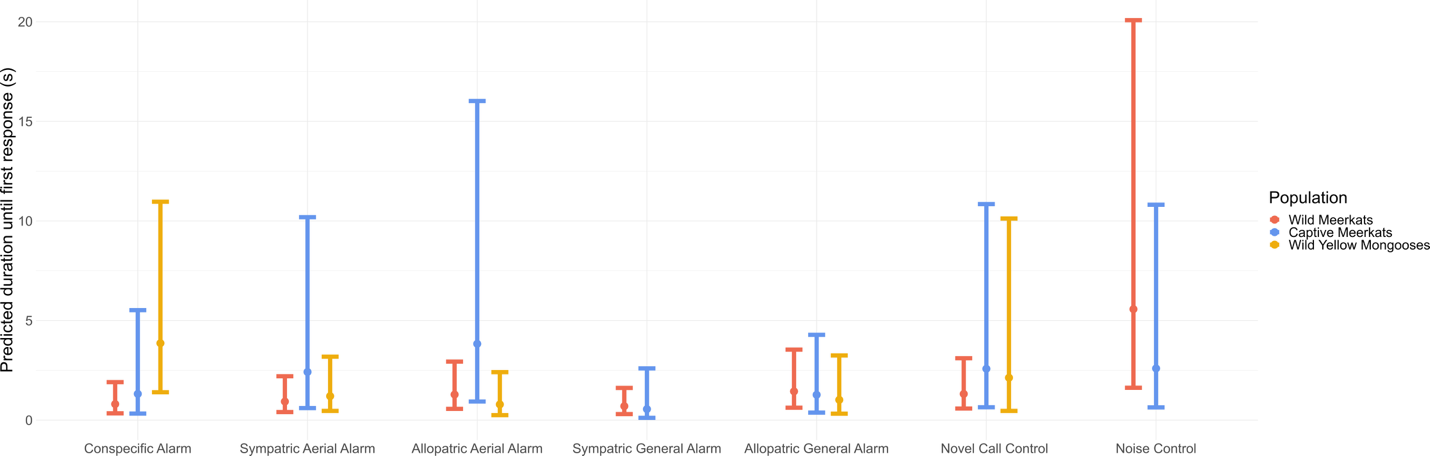

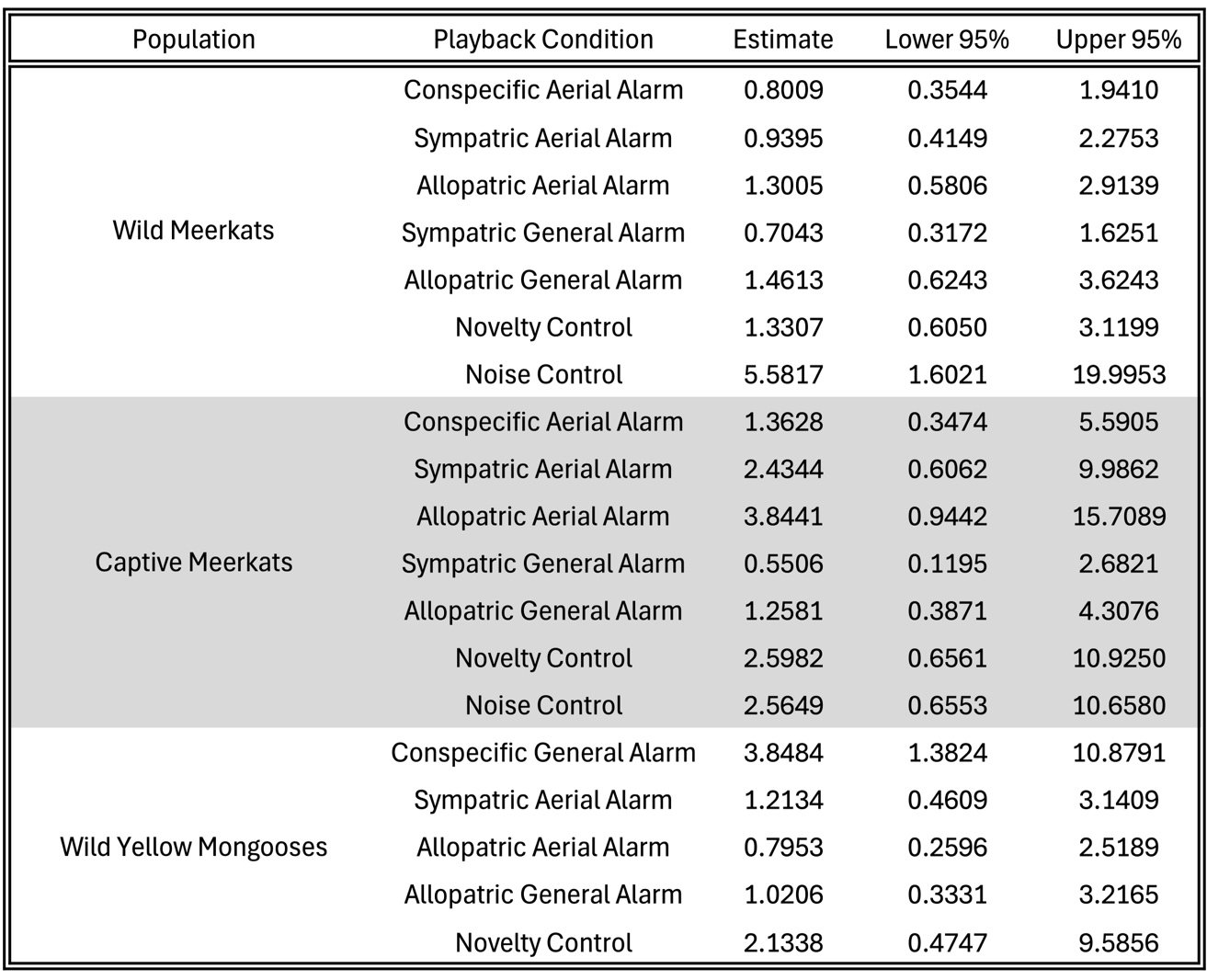
**

**Figure S2.** **Predicted duration until first response as a response to conspecific alarm calls and heterospecific aerial and general alarm calls, produced by either sympatric or allopatric species in wild (red) and captive meerkats (blue) and wild yellow mongooses (yellow).** Each playback condition shows the mean (central dot) and the 95% credible interval (edge lines).

**
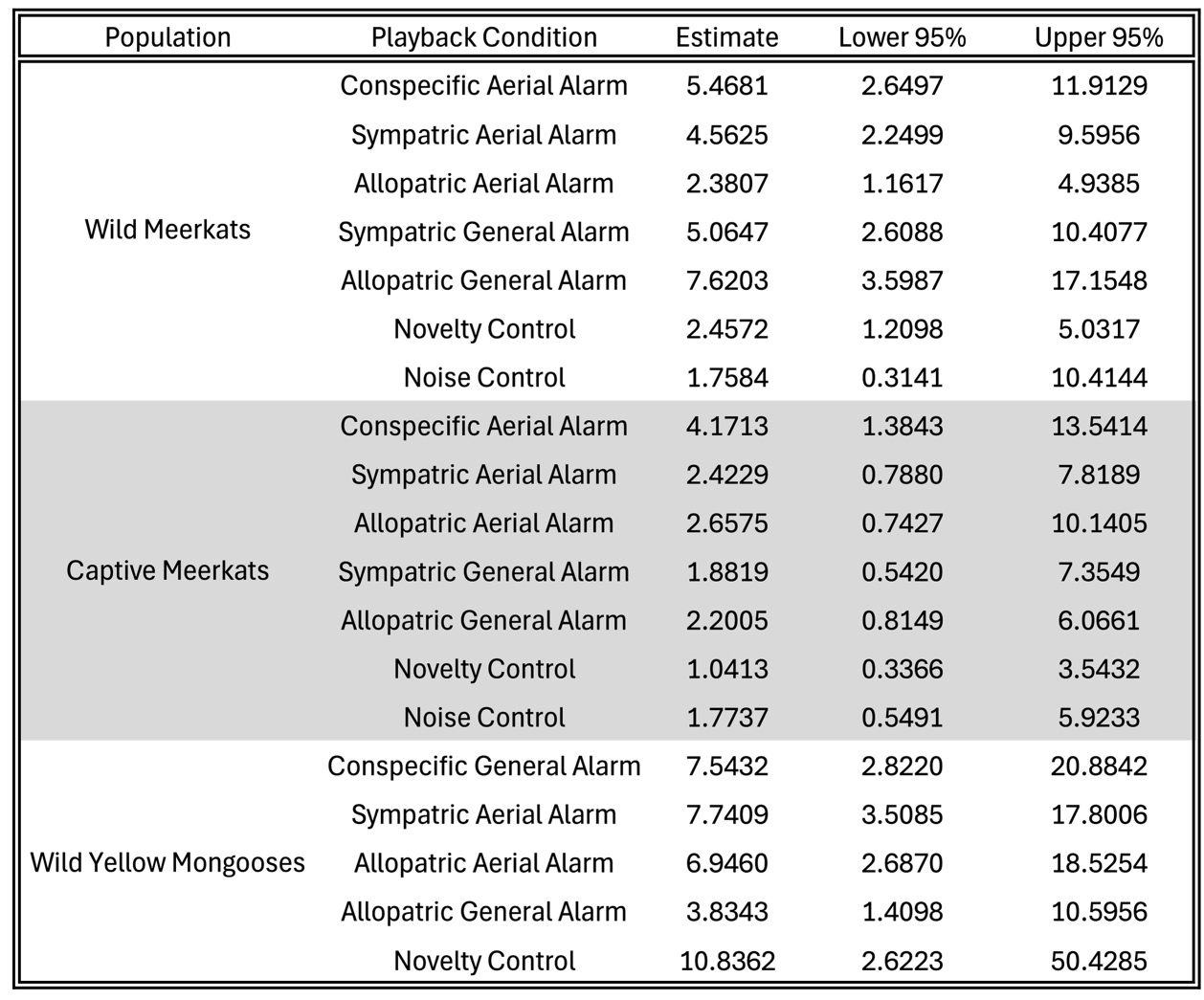
Table S8. Predicted duration of the first gaze event as a response to conspecific alarm calls and heterospecific aerial and general alarm calls, produced by either sympatric or allopatric species in wild and captive meerkats and wild yellow mongooses.** For each condition, the estimated probability and 95% credible intervals are provided.

**
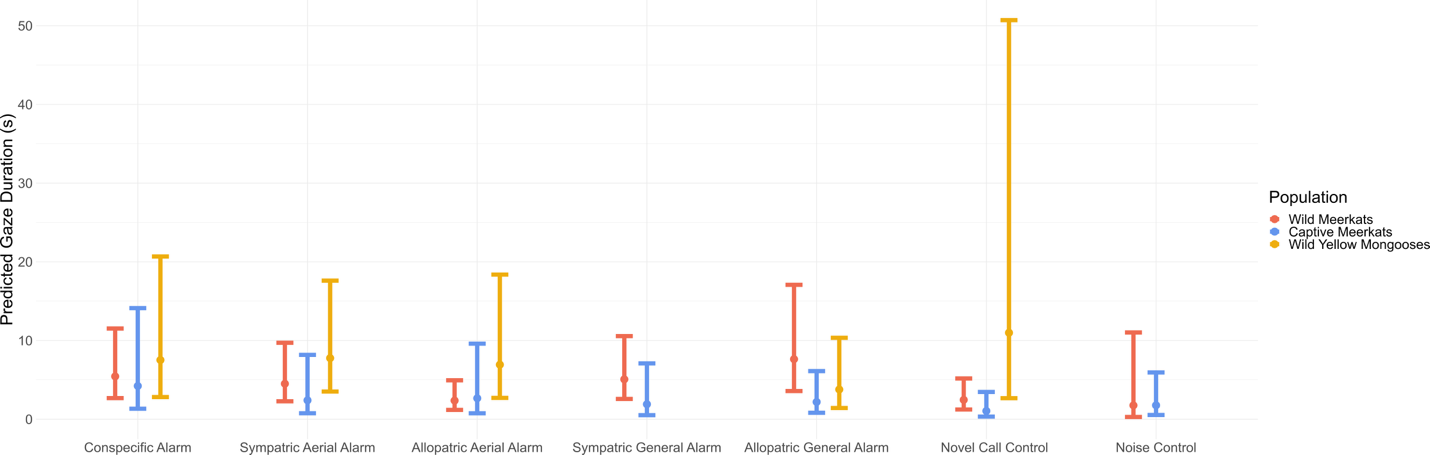
Figure S3. Predicted duration of the first gaze as a response to conspecific alarm calls and heterospecific aerial and general alarm calls, produced by either sympatric or allopatric species in wild (red) and captive meerkats (blue) and wild yellow mongooses (yellow).** Each playback condition shows the mean (central dot) and the 95% credible interval (edge lines).

**
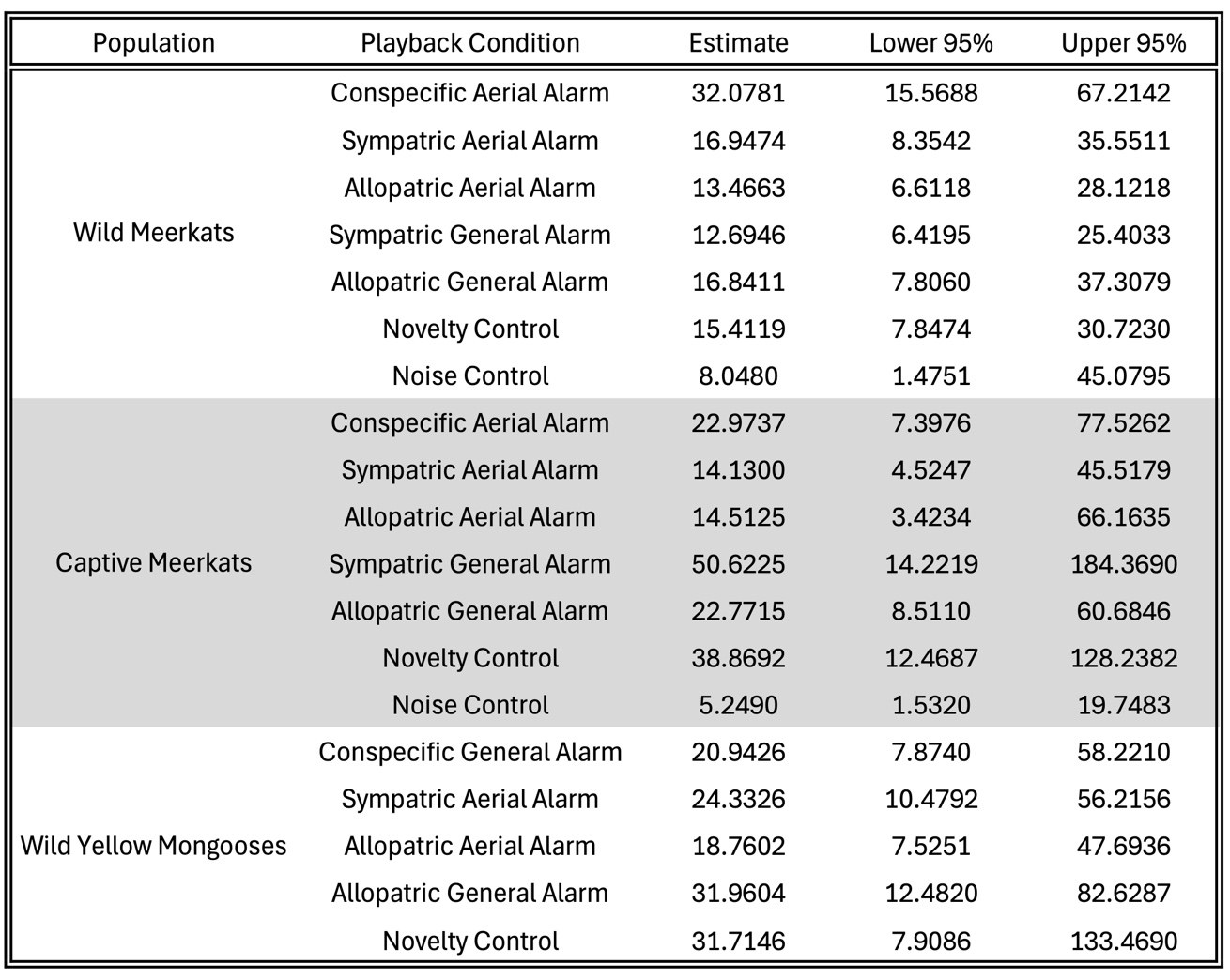
Table S9. Predicted duration until relaxation as a response to conspecific alarm calls and heterospecific aerial and general alarm calls, produced by either sympatric or allopatric species in wild and captive meerkats and wild yellow mongooses.** For each condition, the estimated probability and 95% credible intervals are provided.

**
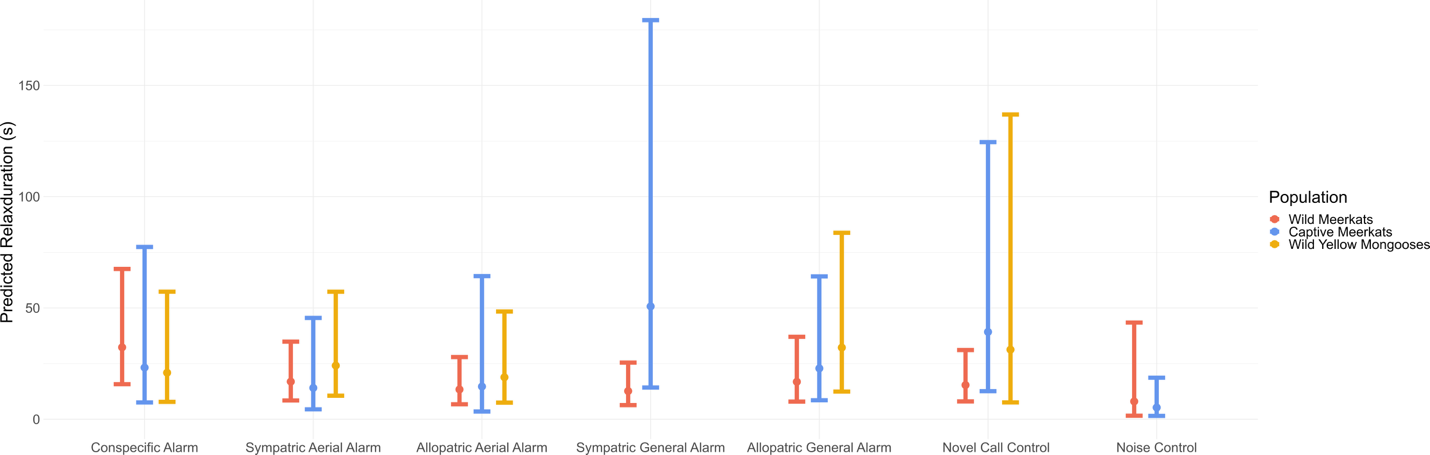
**

**Figure S4. Predicted duration until relaxation as a response to conspecific alarm calls and heterospecific aerial and general alarm calls, produced by either sympatric or allopatric species in wild (red) and captive meerkats (blue) and wild yellow mongooses (yellow).** Each playback condition shows the mean (central dot) and the 95% credible interval (edge lines).

**
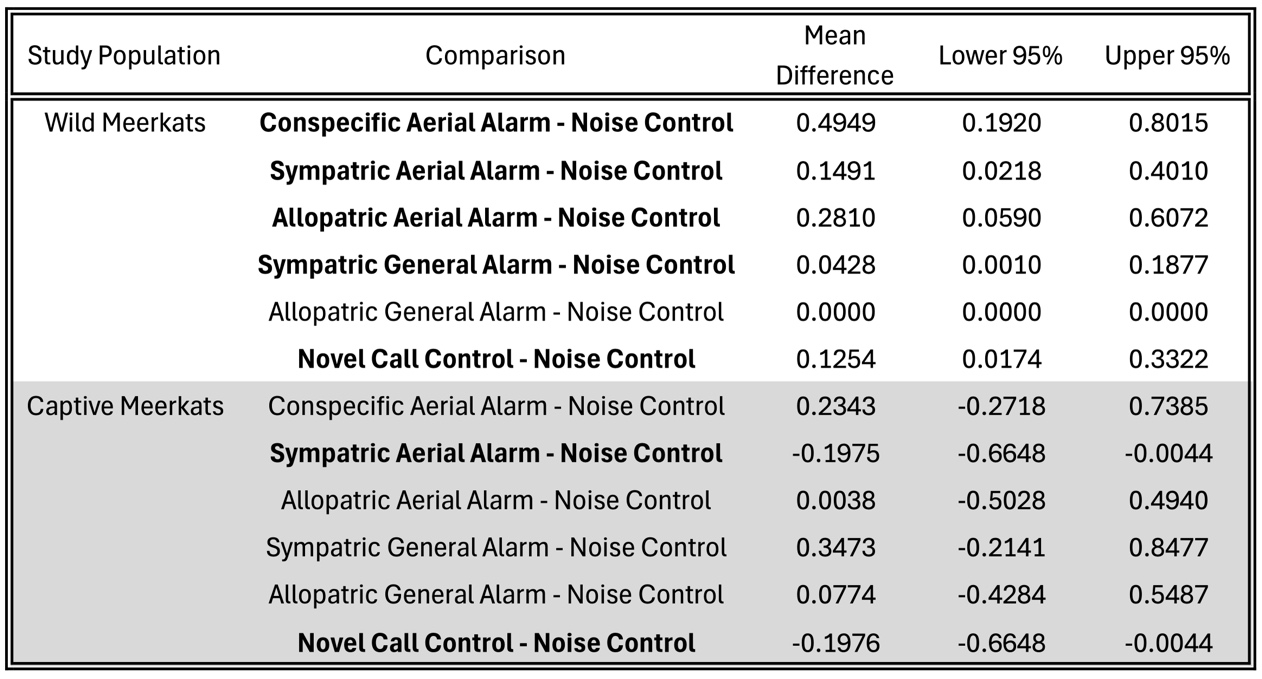
Table S10. Contrasts in the probability to flee between the noise control and all other conditions for wild and captive meerkats.** For each contrast of two conditions, the estimated probability and 95% credible intervals are provided. Comparisons showing a robust difference are indicated in bold.

**
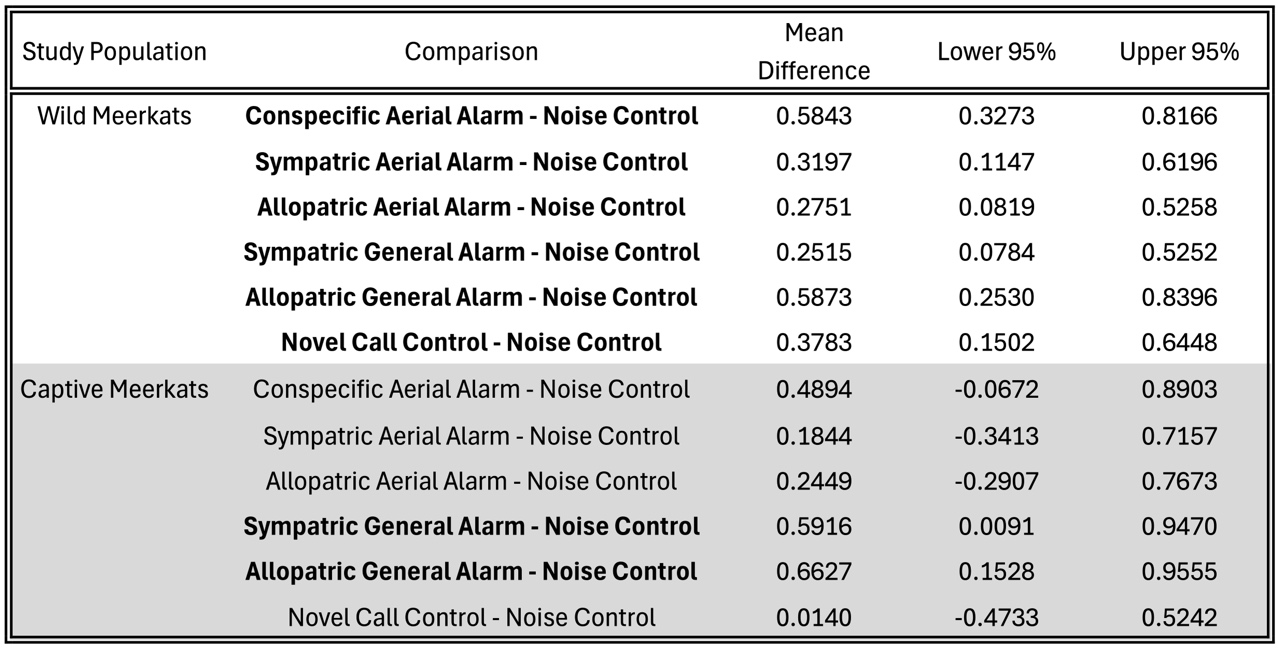
Table S11. Contrasts in the probability to look up into the sky between the noise control and all other conditions for wild and captive meerkats.** For each contrast of two conditions, the estimated probability and 95% credible intervals are provided. Comparisons showing a robust difference are indicated in bold.

**
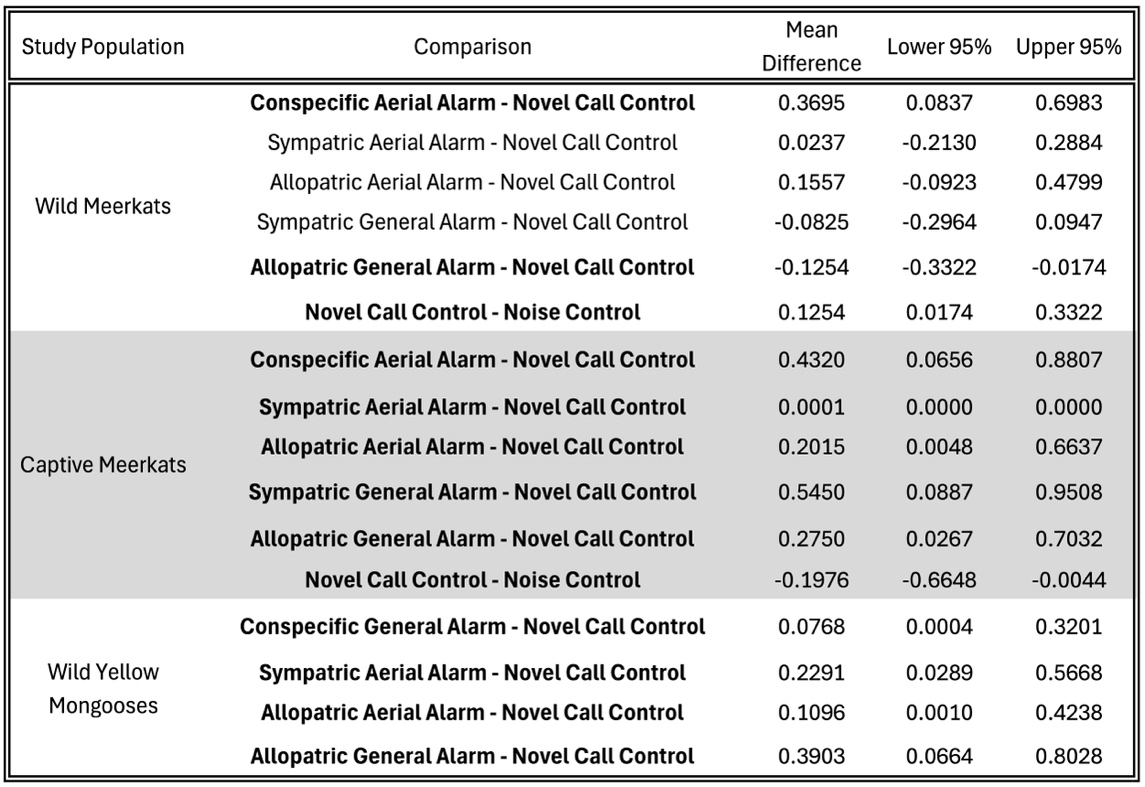
Table S12. Contrasts in the probability to flee between the novel call control and all other conditions for each species.** For each contrast of two conditions, the estimated mean difference in the probability and 95% credible intervals are provided. Comparisons showing a robust difference are indicated in bold.

**
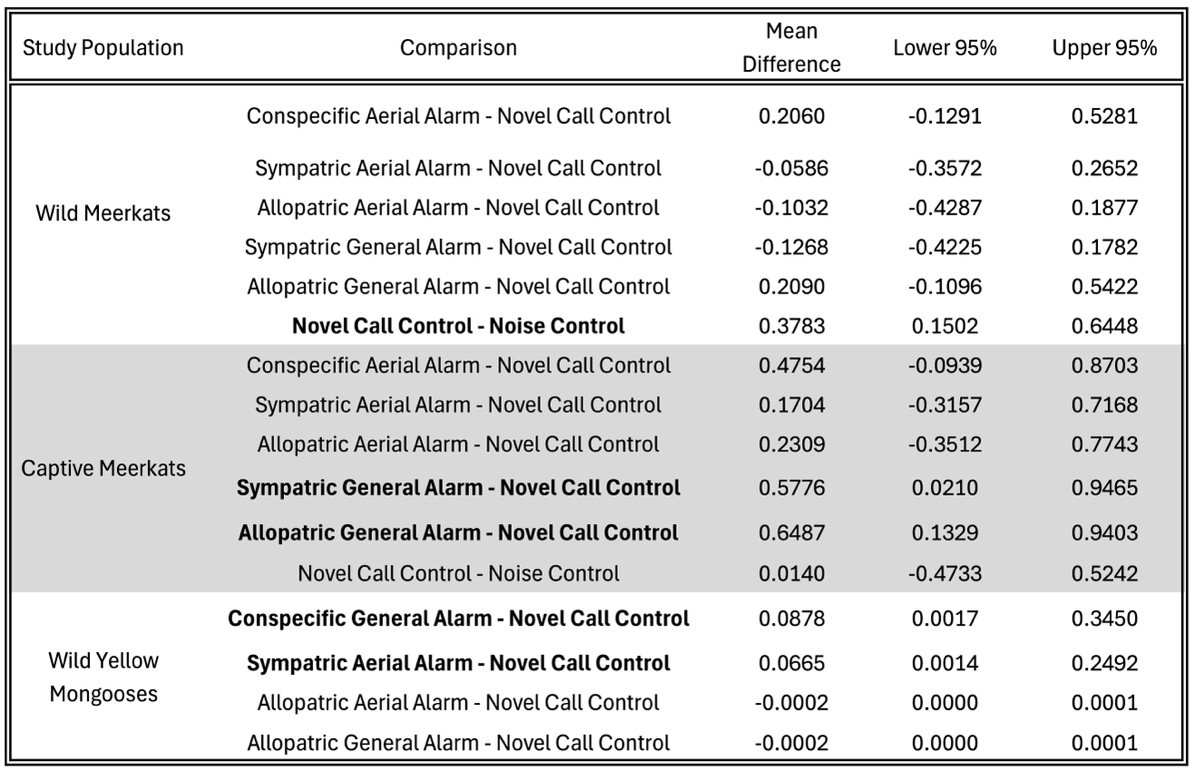
Table S13. Contrasts in the probability to look up into the sky between the novel call control and all other conditions for each species.** For each contrast of two conditions, the estimated mean difference in the probability and 95% credible intervals are provided. Comparisons showing a robust difference are indicated in bold.

**Table S14. Contrasts in the probability to flee between conspecific and heterospecific alarm calls for each species.** For each contrast of two conditions, the estimated mean difference in the probability and 95% credible intervals are provided. Comparisons showing a robust difference are indicated in bold.

**
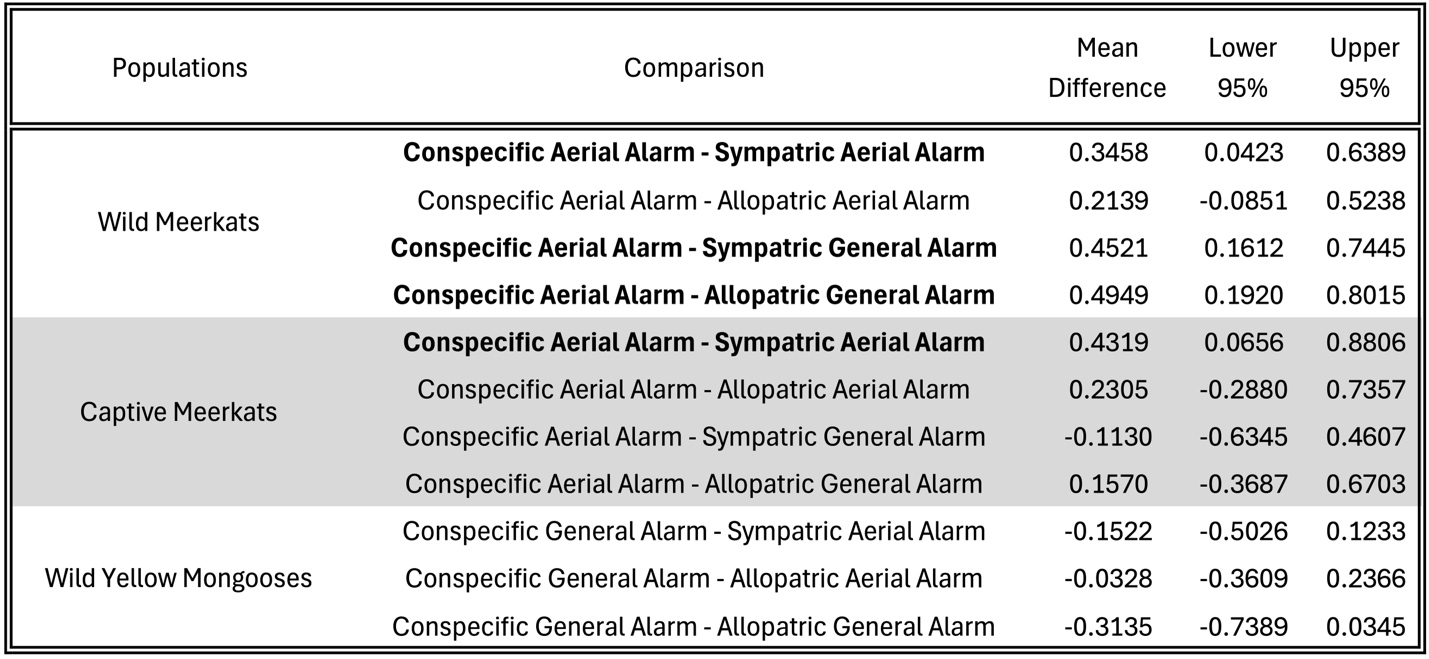
**

**
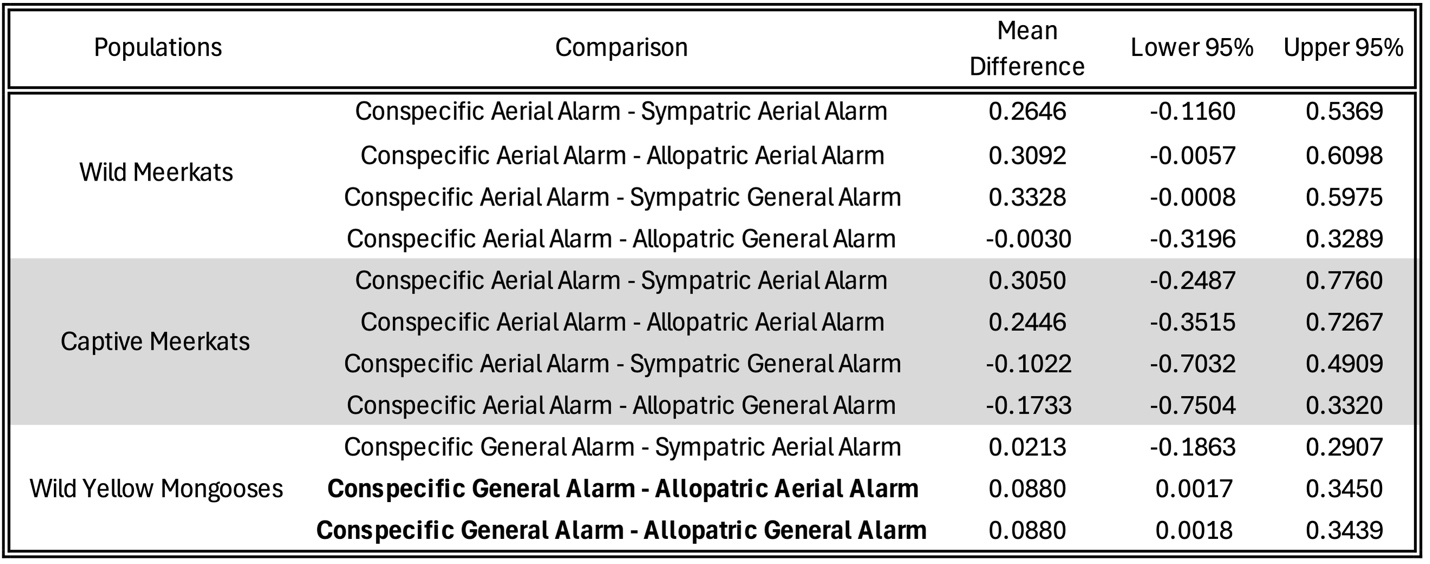
Table S15. Contrasts in the probability to look up into the sky between conspecific and heterospecific alarm calls for each species.** For each contrast of two conditions, the estimated mean difference in the probability and 95% credible intervals are provided. Comparisons showing a robust difference are indicated in bold.

**
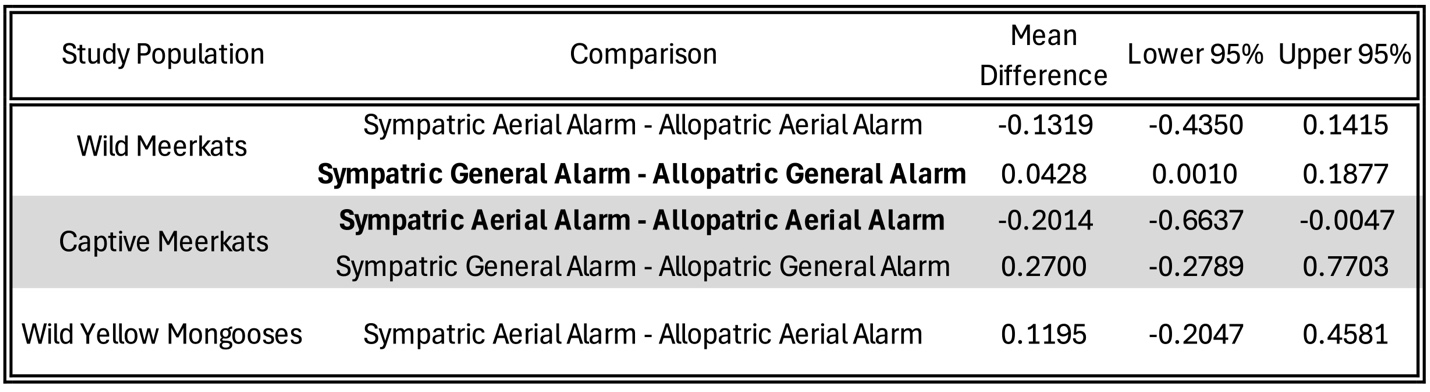
Table S16. Contrasts in the probability to flee between heterospecific alarm calls produced by either sympatric or allopatric species for wild and captive meerkats and wild yellow mongooses.** For each contrast of two conditions, the estimated mean difference in the probability and 95% credible intervals are provided. Comparisons showing a robust difference are indicated in bold.

**Table S17. Contrasts in the probability to look up into the sky between heterospecific alarm calls produced by either sympatric or allopatric species for wild and captive meerkats and wild yellow mongooses.** For each contrast of two conditions, the estimated mean difference in the probability and 95% credible intervals are provided. Comparisons showing a robust difference are indicated in bold.

**
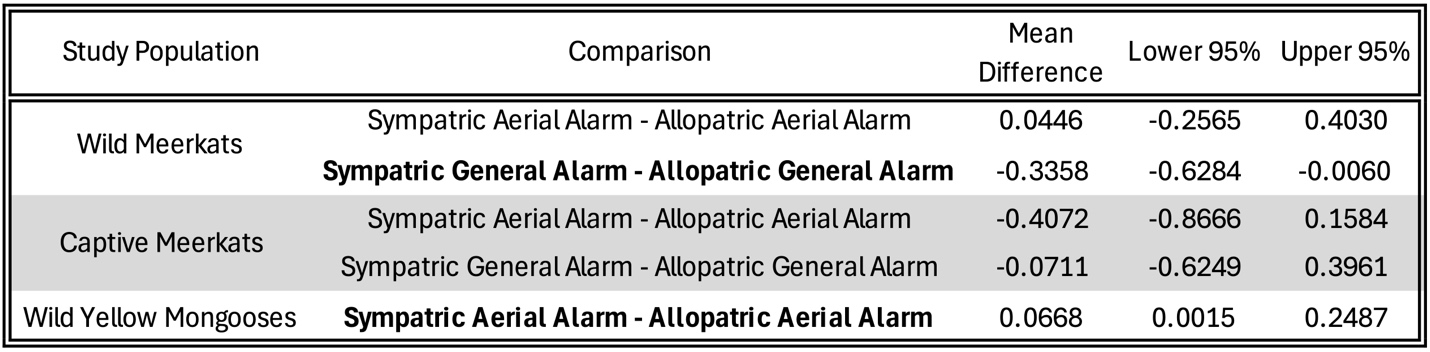
**


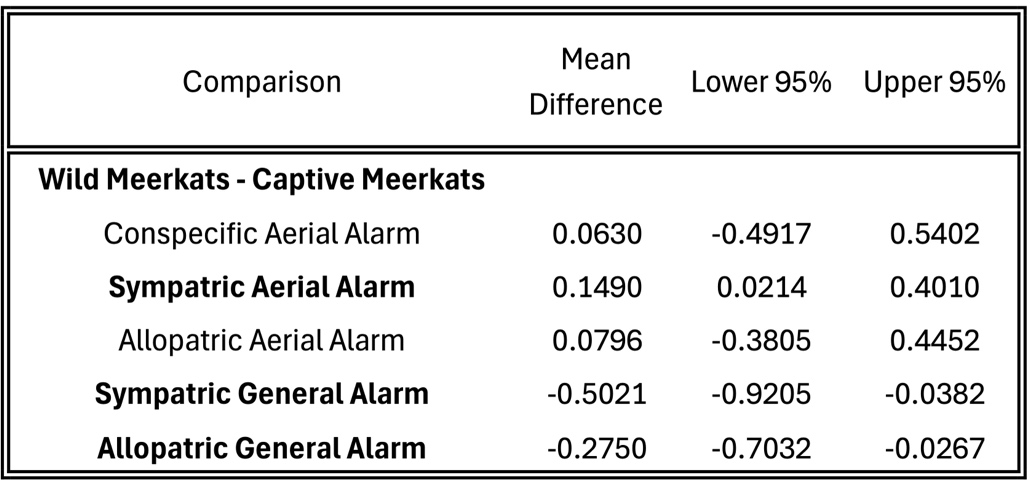
**Table S18. Contrasts in the probability to flee in wild and captive meerkats for conspecific and heterospecific alarm calls.** For each contrast of two conditions, the estimated mean difference in the probability and 95% credible intervals are provided. Comparisons showing a robust difference are indicated in bold.

**Table S19. Contrasts in the probability to look up into the sky in wild and captive meerkats for conspecific and heterospecific alarm calls.** For each contrast of two conditions, the estimated mean difference in the probability and 95% credible intervals are provided. Comparisons showing a robust difference are indicated in bold.

**
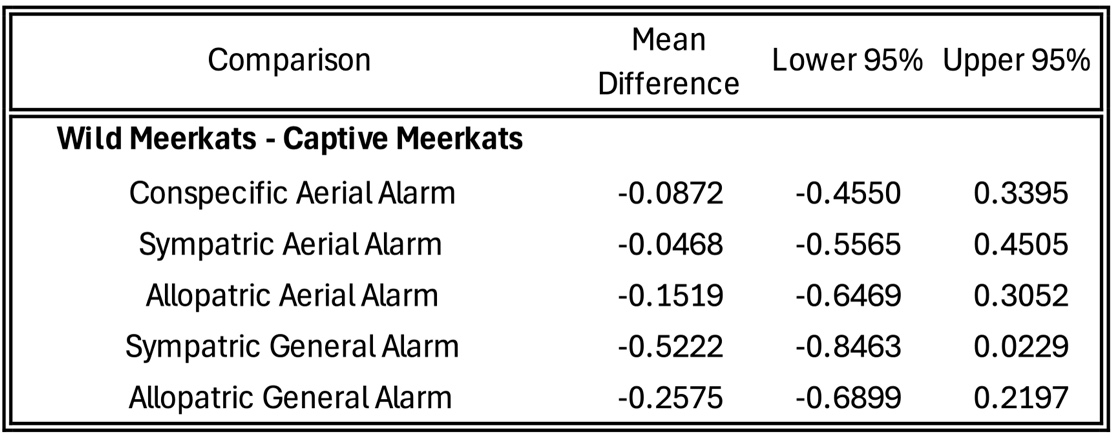
**

**Table S20. Contrasts in the probability to flee between heterospecific aerial and general alarm calls for all species.** For each contrast of two conditions, the estimated mean difference in the probability and 95% credible intervals are provided. Comparisons showing a robust difference are indicated in bold.

**
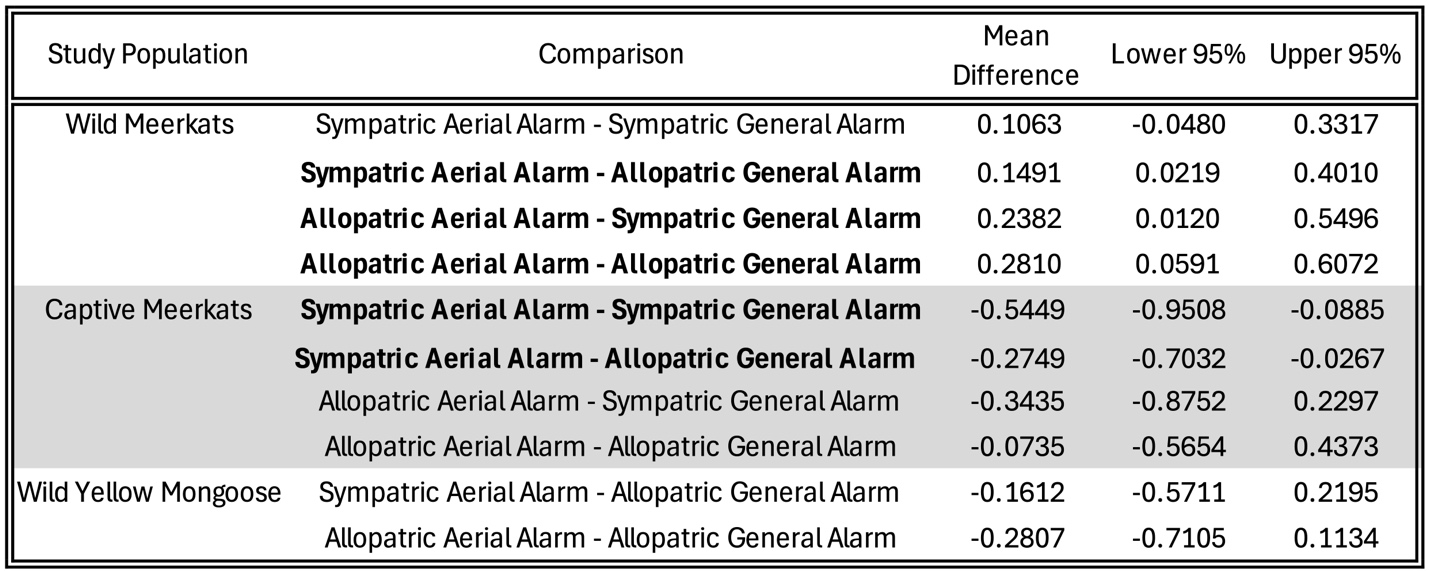
**

**Table S21. Contrasts in the probability to look up into the sky between heterospecific aerial and general alarm calls for all species.** For each contrast of two conditions, the estimated mean difference in the probability and 95% credible intervals are provided. Comparisons showing a robust difference are indicated in bold.

**
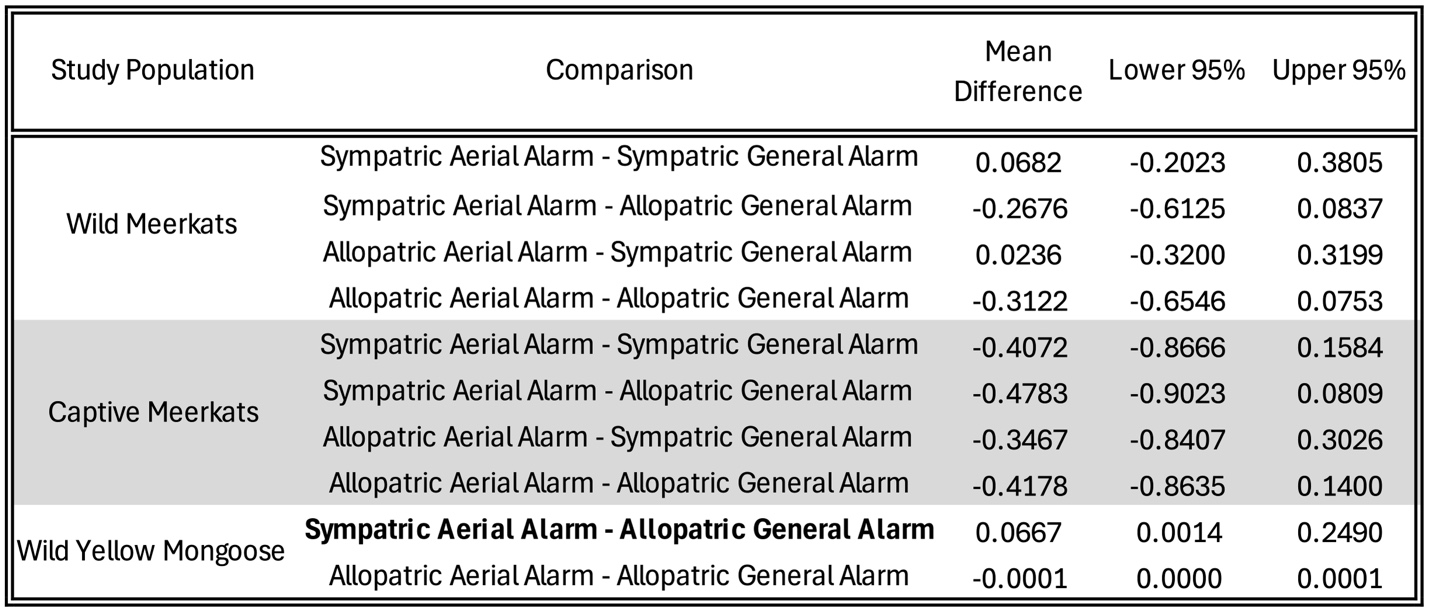
**
